# Breast milk preservation: thermal and non-thermal processes and their effect on microorganism inactivation and the content of bioactive and nutritional compounds

**DOI:** 10.3389/fnut.2023.1325863

**Published:** 2024-02-22

**Authors:** Alejandro Núñez-Delgado, Victoria Margarita Mizrachi-Chávez, Jorge Welti-Chanes, Samantha Thania Macher-Quintana, Cristina Chuck-Hernández

**Affiliations:** ^1^Tecnologico, de Monterrey, School of Engineering and Sciences, Monterrey, Mexico; ^2^Tecnologico de Monterrey, Institute for Obesity Research, Monterrey, Mexico

**Keywords:** maternal milk, expressed breast milk, neonatal nutrition, non-thermal treatments, milk conservation, bioactive compounds, extended breastfeeding

## Abstract

Human Breast Milk (HBM) is widely acknowledged as the best nutritional source for neonates. Data indicates that, in 2019, 83.2% of infants in the United States received breast milk at birth, slightly reducing to 78.6% at 1 month. Despite these encouraging early figures, exclusive breastfeeding rates sharply declined, dropping to 24.9% by 6 months. This decline is particularly pronounced when direct breastfeeding is challenging, such as in Neonatal Intensive Care Units (NICU) and for working mothers. Given this, it is vital to explore alternative breast milk preservation methods. Technologies like Holder Pasteurization (HoP), High-Temperature Short-Time Pasteurization (HTST), High-Pressure Processing (HPP), UV radiation (UV), and Electric Pulses (PEF) have been introduced to conserve HBM. This review aims to enhance the understanding of preservation techniques for HBM, supporting the practice of extended exclusive breastfeeding. It explicitly addresses microbial concerns, focusing on critical pathogens like *Staphylococcus aureus*, *Enterococcus*, *Escherichia coli*, *Listeria monocytogenes*, and Cytomegalovirus, and explores how various preservation methods can mitigate these risks. Additionally, the review highlights the importance of retaining the functional elements of HBM, particularly its immunological components such as antibodies and enzymes like lysozyme and Bile Salt Stimulated Lipase (BSSL). The goal is to provide a comprehensive overview of the current state of HBM treatment, critically assess existing practices, identify areas needing improvement, and advocate for extended exclusive breastfeeding due to its vital role in ensuring optimal nutrition and overall health in infants.

## Introduction

**Figure 1:**
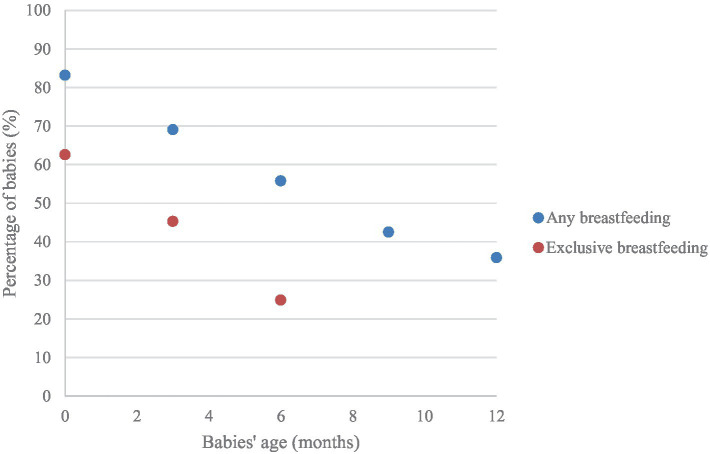
Percentage of 2019-born children who received any or exclusive Human Breast Milk (HBM) for the first 12 months of their lives (5).

While Human Breast Milk (HBM) is often endorsed as the gold standard for neonatal nutrition (1–4), the 2022 Breastfeeding Report Card from the Centers for Disease Control and Prevention (5) reported that the majority (83.2%) of infants born during 2019 in the US began receiving breast milk, and 78.6% were still receiving breast milk at 1 month; however, at 6 months, only 24.9% of infants were exclusively breastfed, while 55.8% occasionally drank breast milk (Figure 1). Also, data reported by UNICEF points out that, according to 2017 data, only 41%–64% of babies were still breastfed at 2 years old (6). These trends have diverse implications, ranging from an increased risk of obesity in the child to various physical and emotional challenges for the mother (7–9).

To address the decline in breastfeeding, it is crucial to comprehend the underlying reasons. While there are numerous factors at play, two prevalent situations arise where direct breastfeeding becomes challenging: (1) When a neonate born preterm requires being introduced to a Neonatal Intensive Care Unit (NICU) (10), (2) Working mothers (representing more than half of mothers, in the US), must go outside the home without their babies, to continue with their professional life (9).

In both scenarios, the core requirement remains consistent: since these babies cannot be breastfed, alternatives must be sought to provide them with HBM when their mothers are not present. Technologies like Holder Pasteurization (HoP), High-Temperature Short-Time (HTST) Pasteurization, High-Pressure Processing (HPP), Ultraviolet (UV) radiation, and Pulsed Electric Fields (PEF) emerge as viable milk preservation methods, each presenting its own set of benefits and limitations.

Considering these factors, this review delves into characterizing HBM as a living fluid, emphasizing its relevance for individual children and society at large. From this perspective, we review the effects of the preservation treatments on the milk’s microbiological, bioactive, and nutritional profiles. The aim is to identify viable alternatives that facilitate prolonged lactation, enabling newborns to be fed exclusively on high-quality HBM for the first 6 months and continue for 12 months, as recommended by major US medical organizations (11), ensuring that infants receive optimal nutrition even when not in immediate proximity to their mothers.

## Breast milk

### Generalities

Human Breast Milk is a complex fluid [sometimes called a biological system (12) and a living tissue], naturally produced by women (13). HBM comprises nutritional and bioactive components that constantly interact with each other (12). Such intricate composition ensures that infants receive optimal nourishment (14). Because of this, organizations like WHO advocate exclusive breastfeeding for at least the initial 6 months of newborns’ lives (15).

**Figure 2:**
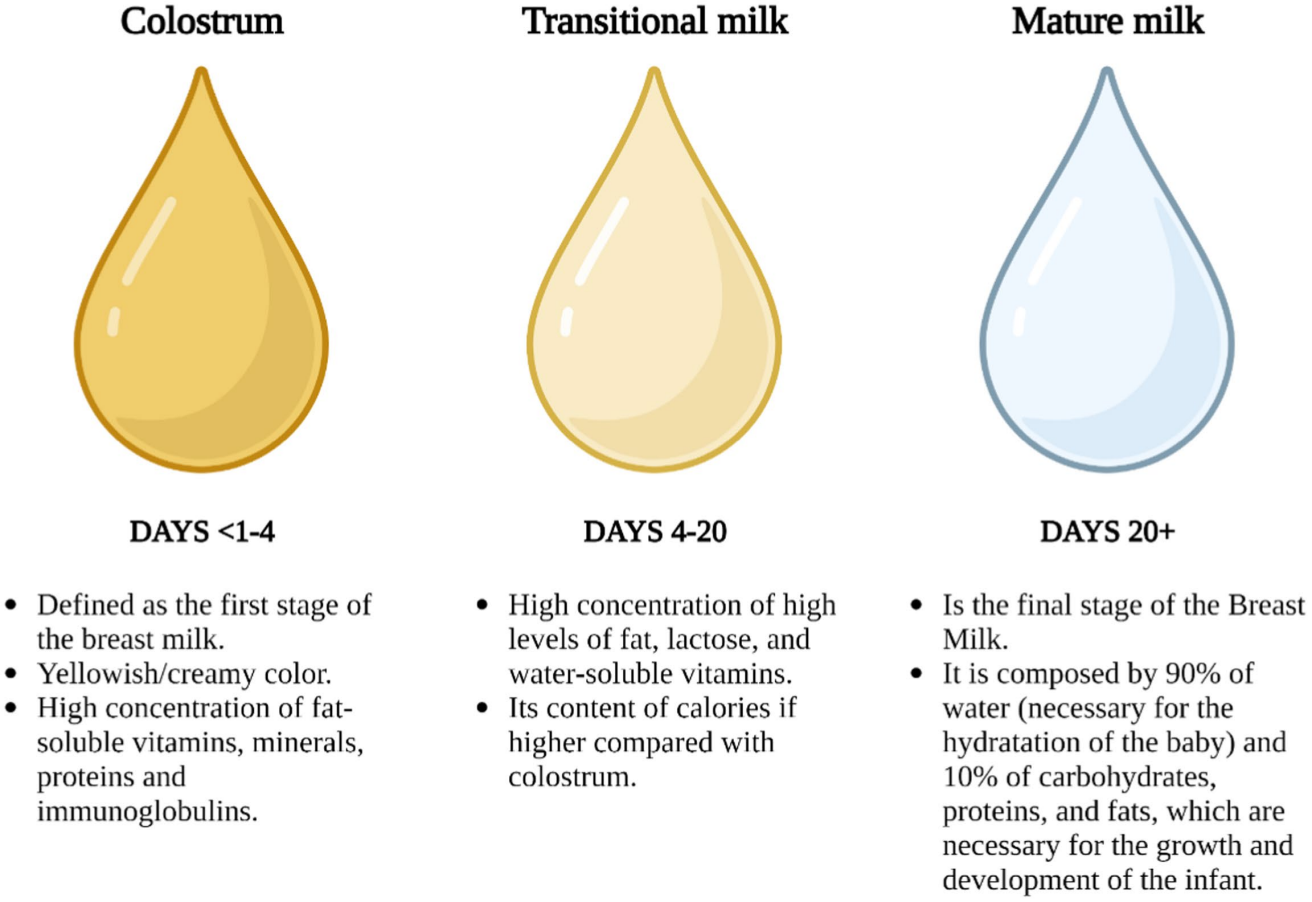
Stages of HBM in the function of periods (16, 17), created with BioRender.com.

While the HBM concept may seem obvious, classifying it poses challenges. This is primarily because (1) its composition constantly adapts to meet the evolving needs of the newborn, and (2) the dynamics of its constituents are influenced by various factors related to the mother, the baby, and the environment, including variables like geographic location and the mother’s diet (12, 13). With these complexities in mind and with a degree of generalization, the primary phases of HBM can be classified as colostrum, transitional milk, and mature milk, with their primary characteristics illustrated in Figure 2.

This classification offers an introductory insight into the complexity of HBM, to understand its significance in neonatal nutrition and (13) the indispensable role that this fluid plays in newborn feeding.

### Composition

#### Bioactive components in HBM

**Figure 3:**
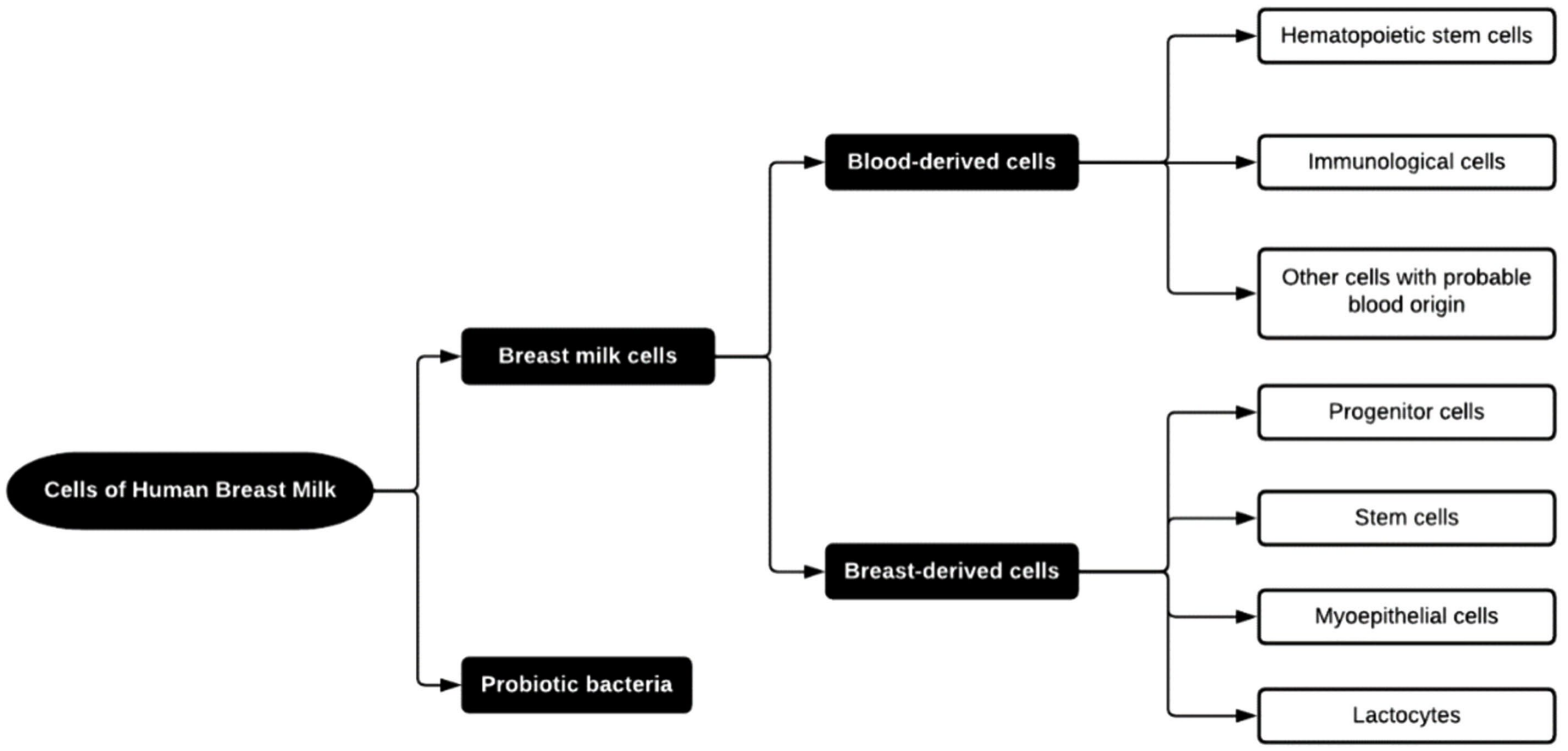
Cells found in HBM (13).

While inherent variability exists, bioactive compounds in HBM can be generally categorized into (1) growth and immunological factors and (2) cellular components, which notably include beneficial bacteria, along with immune, epithelial, and stem cells (13). A summary of these components is shown in Figure 3.

The emphasis on immunological factors, specifically antibodies, is paramount within bioactive components. Immunoglobulins are prominent representatives of this category. They are present mainly in the form of secretory Immunoglobulin A (sIgA) (hypothesized as an essential protective agent in breast milk) and secretory Immunoglobulin G (sIgG). These components play a crucial role in safeguarding the infant from infections during the maturation of its immune system. Breastfeeding has been shown to reduce the incidence of respiratory tract infections by 63% (18, 19). Remarkably, the content of immunoglobulins decreases as the lactation progresses. This trend can be attributed to (1) the increased autonomy of the infant’s immune system, reducing the need for external support, and (2) a decline in the newborn’s ability to absorb whole proteins through the gut (18).

HBM comprises both immunological and non-immunological cells. Concerning the former, leucocytes represent less than 2% of all cell content in the mature milk of healthy mothers. Their role is to bestow immunocompetence upon the newborn, and some hypotheses suggest a role in protecting the mammary gland against infections (13).

**Figure 4:**
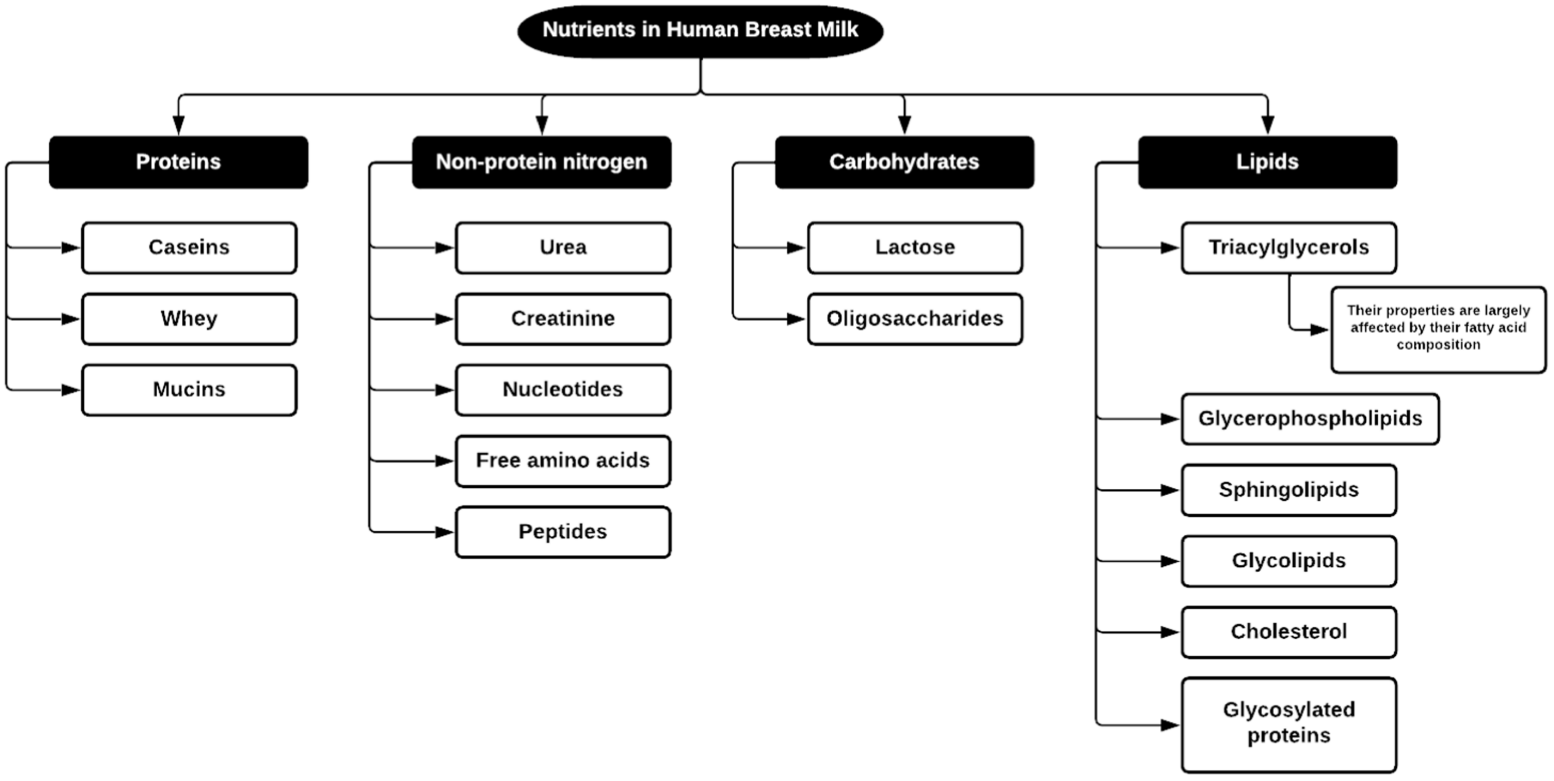
Nutrients found in HBM (20).

Non-immunological cells in HBM can be classified as lactocytes (responsible for the secretion of milk), myoepithelial cells (coming from ducts and alveoli from the mammary gland), progenitor cells, and stem cells, present in a heterogeneous mixture (Figure 4). Notably, myoepithelial cells and their precursors constitute around 98% of the non-immune cell types in healthy HBM. Their predominance is owed to their fundamental role in building the smooth muscle fibers surrounding the alveoli. Their importance is further underscored by their specific function of facilitating milk flow into the milk ducts (13). While existing studies provide insights, it is pertinent to acknowledge the need for further research to elucidate the potential benefits of non-immune cells in infant health.

#### Nutritional components of HBM

As mentioned before, the nutrient profile of HBM can differ based on several factors, notably the duration post-birth. Figure 4 illustrates the classification of nutrients found in HBM. Subsequently, we delve into each nutrient cluster’s role in neonatal development.

##### Proteins

Proteins are integral components of HBM, highlighted by (1) the variety of their functions, since they play a role in stimulating the absorption of other nutrients, modulating the growth and body composition of newborns, promoting gut development, and in antimicrobial and immunomodulatory activities, among other purposes, and (2) for its broad types; it is reported that HBM can contain more than 400 types of proteins (18, 20).

Lactocytes synthesize between 80% and 90% of proteins in HBM, with the remainder sourced from maternal circulation via transcytosis, subsequently entering the breast duct lumen (18).

As outlined in Figure 4, proteins are primarily classified into (1) casein proteins, existing as α-, β- and κ-casein in micelles, forming a colloidal suspension with κ-casein acting as a stabilizer; (2) whey proteins, soluble in milk, prominently including α-lactalbumin, lactoferrin, immunoglobulin S (IgS), serum albumin and lysozyme, and (3) mucins proteins, localized in the membrane of the fat globules in milk (18).

It is essential to mention that whey proteins such as lactoferrin and lysozyme are bioactive proteins that protect the infant against pathogens. First, lactoferrin (a highly glycosylated protein) can affect iron-dependent microorganisms because of its ability to decrease iron availability and disrupt their membranes (21, 22) and has antiviral effects against diverse viruses, including SARS-CoV-2 (23–25). As for Lysozyme, it also possesses an antimicrobial effect, but its range of action focuses on improving human-resident bifidobacteria by excluding the non-human types from the infant’s system (20, 26).

##### Non-protein nitrogen

This category, detailed in Figure 4, accounts for approximately 25% of all the nitrogen present in HBM (18). Although this fraction has been little studied, research underscores its contributions to metabolic mediation, enzymatic activity, and promoting gut and microbiota development (18).

##### Carbohydrates

Lactose, a disaccharide comprising glucose and galactose, dominates the carbohydrate content in HBM. HBM has the highest lactose concentration compared to other mammals, mainly attributed to the highest energy demand for human brain development (18). Furthermore, as a galactose source, lactose is pivotal for the maturation of the central nervous system (20).

Human Milk Oligosaccharides (HMOs) are another vital carbohydrate component in HBM. While not directly digested by newborns, they play an essential role in nourishing the gut microbiota (18).

##### Human Milk Oligosaccharides (HMOs)

HMOs are complex molecules composed of 3–22 monomeric units of saccharides per molecule. Their structural building blocks consist of five monomers: L-fucose, D-glucose, D-galactose, N-acetylglucosamine, and N-acetylneuraminic acid, which vary in orientation and sequence of bonding (18, 27).

Several unique properties distinguish HMOs from other carbohydrates. They are the third most abundant component in HBM, with concentrations reaching up to 12.9 g/L in mature milk (18). Functionally, HMOs are essential for developing the infant’s gut microbiota and encouraging the growth of beneficial gut bacteria (serving as growth substrates), such as *Bifidobacterium infantis* (28). This enhancement helps to mitigate the growth of pathogenic bacteria and establishes a protective barrier against neonatal diarrheal infections. This protective mechanism stems from HMOs’ ability to mimic intestinal cell carbohydrates, which some pathogens latch onto. HMOs intercept these pathogens by acting as decoys, preventing them from infecting epithelial cells (18, 29).

##### Lipids

**Figure 5:**
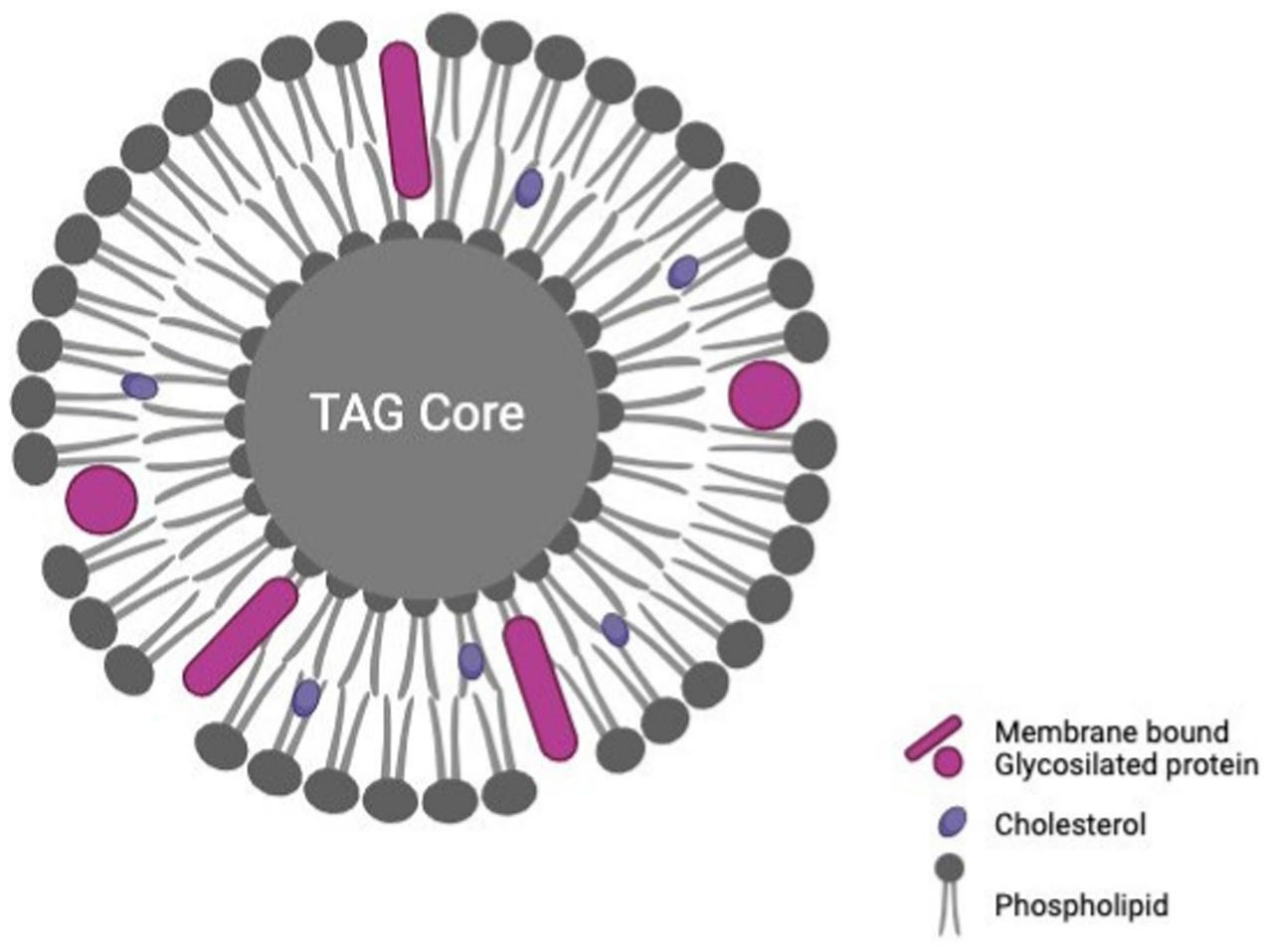
Conformation of a milk fat globule with a triacylglyceride (TAG) core, based on Brink and Lönnerdal (30), created with BioRender.com.

These compounds represent the primary energy source in HBM, accounting for 40%–55% of its total energy, as shown in Table 1 (20). Notably, nearly 98% of the lipids in human milk are triacylglycerides (TAG). The remaining fractions include diacylglycerides, monoacylglycerides, free fatty acids, phospholipids, and cholesterol. These molecules can form emulsions, forming fat globules. Within this structure, phospholipids comprise the protective membrane encapsulating the TAG core, as shown in Figure 5 (18, 30).

**Table 1:** Contribution to energy intake to 1 month of age newborns of compounds found in HBM, based on Mosca and Giannì (20).

Compound	Energy intake (%)
Lipids	44.5 ± 5.2
Carbohydrates	43.9 ± 5.8
Proteins	8.4 ± 1.0

Beyond their primary energy-providing role, lipids in HBM also play a vital role in infant development. The concentration of long-chain polyunsaturated fatty acids (LC-PUFAs) in breast milk, such as DHA and arachidonic acid (ARA), positively correlates with infants’ cognitive development and visual acuity. Also, they serve as a source of essential nutrients, such as lipid-soluble vitamins, polyunsaturated fatty acids, and complex lipids. Furthermore, they play a crucial role in the myelination of the central nervous system, foster the development of the gastrointestinal tract, and offer protection against infections on the mucosal surface (18, 20, 31).

Another significant lipid fraction in HBM is glycolipids, which play a specific role in infant development. Recent studies indicate that gangliosides, a type of glycolipid, contribute to the maturation of the immune response and offer protection against allergies. They also have beneficial roles in antibacterial, anti-inflammatory, and prebiotic responses (32).

### Significance of breastfeeding: implications for newborns, mothers, and public health

HBM has been frequently cited as: “the only food that meets all the nutritional requirements of infants and provides optimal adaptation, somatic growth, maturation, and development” (12, 33). The evidence highlights that the first one hundred days post-conception is the most critical period for laying the foundation of lifelong health (34). This underlines the profound impact of breastfeeding on the health trajectories of infants and mothers, immediately and in the long run, and extends its significance to broader public health contexts.

For infants, the benefits are clear. In the immediate term, research suggests a discernible decrease in mortality rates during the initial years of life of breastfed newborns (34). Additionally, HBM has been highly effective in guarding against infectious gastroenteritis, particularly in developing countries, while bolstering immune system maturation (33). Over the long term, HBM plays dual roles: (1) fostering the growth and stability of the microbiome, which in turn may mitigate risks associated with chronic diseases like obesity, diabetes, heart disease, and cancer (34), and (2) facilitating the maturation of the central nervous system, which correlates with enhanced cognitive performance (studies have indicated a potential 2%–3% increase in intelligence quotient and improved academic outcomes) (12, 35).

For mothers, breastfeeding offers protective benefits against certain cancers potentially due to its ovulation-suppressing effects (34, 36). Moreover, research indicates that compared with their non-lactating counterparts, breastfeeding mothers often require fewer medical consultations (23), a testament to the health benefits summarized in Table 2 (37).

**Table 2:** Breastfeeding benefits for mothers, based on del Ciampo and del Ciampo (37).

Short-term benefits	Long-term benefits
Reduced probabilities of:	Reduced probabilities of:
Bleeding	Cancer (endometrium, breast, and ovarian)
Infection	Endometriosis
Adiposity and weight	Chronic diseases, such as diabetes, high blood pressure, and cardiovascular diseases
Postpartum depression	Rheumatoid arthritis
Stress and anxiety	Alzheimer disease
Get pregnant immediately	–

Breastfeeding offers significant health benefits as a form of preventive medicine, nurturing a healthier population less prone to chronic diseases responsible for 60% of deaths globally (38). Emphasizing its health impact could revolutionize public health, akin to the transformative change seen almost 60 years ago regarding smoking, after the US Surgeon General’s report on smoking worldwide. Supporting every mother’s ability to breastfeed, as urged in 2011, the Surgeon General’s Call to Action is crucial for global health improvement (34, 39).

### Why preserve Human Breast Milk?

In response to the US Surgeon General’s call, the treatment of HBM emerges as a pivotal alternative for mothers desiring to breastfeed but facing constraints. This article will delve into two primary scenarios underscoring this need.

First is the case of newborns, mainly preterm infants, temporarily deprived of direct maternal contact in the NICU. In such cases, Donated Breast Milk (DBM) would be the preferred option to meet the newborn’s nutritional needs (40). Given the vulnerable and underdeveloped immune systems of preterm infants, they are inherently at higher risk of infections (41). Therefore, to ensure its microbiological safety, DBM is typically processed in a Human Milk Bank (HMB), a specialized facility designed for this purpose. The Holder Pasteurization Process, endorsed by the Human Milk Banking Association of North America (HMBANA), defines the standard protocol (42). This method, while ensuring microbial safety, can compromise vital compounds like enzymes, immunoglobulins, and growth factors. Consequently, thermal and non-thermal techniques are being explored as alternatives for ensuring microbial safety and nutrient preservation in HBM.

The second scenario regards the challenge of nursing for working mothers. Data from 2011 (9, 43) indicates that a significant percentage (64%) of American women with children below 6 years and 56% with infants under 1 year were employed outside their homes. This pattern has persisted globally well into the last decade (9). Undoubtedly, this trend influences breastfeeding duration. Studies highlight a noteworthy difference in the rates of lactation continuation between working mothers and their stay-at-home counterparts, with the former group showing at least a 9% decline by the six-month mark (9, 44). This disparity stems from several factors, including workplace conditions and lack of technology that supports milk extraction and preservation, leading many working mothers to preemptively view workplace breastfeeding as discouraging. Often, balancing workplace demands with maternal responsibilities becomes overwhelming, breastfeeding is the first thing they usually abandon, despite its benefits for both mother and newborn (9).

Given this context, devising solutions to aid mothers to continue breastfeeding comfortably and sustainably, without negatively affecting their employment, becomes highly relevant. Among the innovative avenues explored are emerging food preservation technologies like HPP, UV radiation, and PEF. Not only do these techniques ensure the microbiological safety of HBM until consumption, but they also ensure it retains essential nutrients and bioactive compounds pivotal for newborn development. The promise of these alternatives is further buoyed by findings suggesting extended lactation durations when workplaces provide suitable facilities for breast milk extraction and preservation technology (9).

In conclusion, exploring viable HBM preservation methods is fundamental for enhancing lactation duration for preterm and term newborns. Such advancements empower those mothers unable to breastfeed directly to persist with lactation, thereby bolstering the health of both mother and child and paving the way for a better-nourished and healthier society. That is why, in the subsequent sections, we provide an overview of various technologies employed for HBM treatment. We will also discuss their impact on nutritional and functional properties and their relevance from a microbiological preservation perspective.

## Thermal vs. non-thermal processes: a comparison of effects in breast milk

Even though several technologies could be applied for HBM treatment, exploring which ones can make milk safe from the microbiological perspective is very important. It is necessary to completely inactivate microorganisms [that is, when viruses are inactivated, and bacterial reduction achieve 5-log reductions and levels below 10 CFU/mL (45, 46)] without compromising the content and activity of bioactive and nutritional compounds crucial for pre-term and term babies’ health. Considering this, a comparative analysis using Tables 3–6 is shown.

**Table 3:** Effect of thermal processes applied for pathogen population reduction on breast milk.

Applied treatment	Process conditions	Microorganism	Log_10_ reductions	Initial load	Units^A-E^	Reference
HoP	62.5°C, 30 min	*Bacillus cereus* (CECT 131)	2.62	10^7^	CFU/mL	(47)
	63 ± 0.5°C, 30 min	Coagulase-negative staphylococci*	Below detection limit	9.8×10^4^	CFU/mL	(48)
	63°C, 30 min. After heating: 4°C	Cytomegalovirus (CMV)	>0.9	3.16×10^3^	PFU/mL	(49)
	62.5°C, 30 min	Cytomegalovirus (CMV) (AD169)	Below detection limit	32	IEA	(50)
	62.5°C, 30 min	Enveloped hepatitis E virus (eHEV)	1.1	6.31×10^4^	FFU/mL	(51)
	62.5°C, 30 min	*Enterococcus faecalis* (ATCC 29212)	>5.1	1.16×10^5^	CFU/mL	(50)
	62.5°C, 30 min. After heating: quickly cooled to 20°C	*Enterococcus faecalis* (PCM 896)	3.92	8.91×10^4^	CFU/mL	(52)
	62.5°C, 30 min. After heating: quickly cooled to 20°C	*Enterococcus faecium* (ATCC 6057)	4.28	7.94×10^4^	CFU/mL	(52)
	63 ± 0.5°C, 30 min	Enterococcus species*	Below detection limit	10^3^	CFU/mL	(48)
	62.5°C, 30 min. After heating: cooled in ice slurry	*Escherichia coli* (ATCC 25922)	≥7	10^8^	CFU/mL	(53)
	62.5°C, 30 min. After heating: quickly cooled to 20°C	*Escherichia coli* (K-12)	Below detection limit	1.05×10^5^	CFU/mL	(52)
	62.5°C, 30 min	HCoV-229E	1.5	10^6^	FFU/mL	(51)
	63°C, 30 min. After heating: 4°C	*Hepatitis A virus* (HAV)	3.1	5.01×10^5^	PFU/mL	(49)
	62.5°C, 30 min	*Klebsiella pneumoniae* (ATCC 700603)	>4.8	5.70×10^4^	CFU/mL	(50)
	62.5°C, 30 min	*Klebsiella pneumoniae* (Kpn 01605)	>4.6	3.60×10^4^	CFU/mL	(50)
	62.5°C, 30 min. After heating: cooled in ice slurry	*Listeria monocytogenes* (ATCC 19115)	≥7	10^8^	CFU/mL	(53)
	62.5°C, 30 min	Non-enveloped hepatitis E virus (neHEV)	1.2	5.01×10^5^	FFU/mL	(51)
	62.5°C, 30 min	*Pseudomonas aeruginosa* (ATCC 27853)	5	10^5^	CFU/mL	(50)
	62.5°C, 30 min. After heating: cooled in ice slurry	SARS-CoV-2	6	10^7^	TCID_50_	(54)
	62.5°C, 30 min	*Serratia marcescens* (Smarc 00697)	>4.9	1.04×10^5^	CFU/mL	(50)
	62.5°C, 30 min. After heating: cooled in ice slurry	*Staphylococcus agalactiae* (ATCC 12927)	≥7	10^8^	CFU/mL	(53)
	62.5°C, 30 min. After heating: cooled in ice slurry	*Staphylococcus aureus* (ATCC 25923)	≥7	10^8^	CFU/mL	(53)
	62.5°C, 30 min	*Staphylococcus aureus* (ATCC 6538)	>4.9	7.90×10^4^	CFU/mL	(50)
	62.5°C, 30 min. After heating: cooled in ice slurry	*Staphylococcus aureus* (ATCC 6538)	≥7	10^8^	CFU/mL	(53)
	62.5°C, 30 min. After heating: quickly cooled to 20°C	*Staphylococcus aureus* (PCM 2054)	Below detection limit	1.20×10^5^	CFU/mL	(52)
	62.5°C, 30 min	*Staphylococcus aureus* sub. Aureus (CECT 976)	3.13	10^7^		(47)
HTST	72°C, 16 s	Bovine Viral Diarrhea Virus (BVDV)	>5.84	6.31×10^7^	TCID_50_	(55)
	72°C, 15 s	*Chronobacter sakazakii* (ATCC 51329)	Below detection limit	1.60×10^6^	CFU/mL	(56)
	62°C, 5 s	Coagulase-negative staphylococci*	4.42	9.80×10^4^	CFU/mL	(48)
	62°C, 5 s	Cytomegalovirus (CMV) (AD169)	Below detection limit	32	IEA	(50)
	62°C, 15 s	Cytomegalovirus (CMV) (AD169)	Below detection limit	32	IEA	(50)
	72°C, 5 s	Cytomegalovirus (CMV) (AD169)	Below detection limit	32	IEA	(50)
	72°C, 15 s	Cytomegalovirus (CMV) (AD169)	Below detection limit	32	IEA	(50)
	87°C, 2 s	Cytomegalovirus (CMV) (AD169)	Below detection limit	32	IEA	(50)
	87°C, 5 s	Cytomegalovirus (CMV) (AD169)	Below detection limit	32	IEA	(50)
	62°C, 5 s	*Enterococcus faecalis* (ATCC 29212)	1.1	1.16×10^5^	CFU/mL	(50)
	62°C, 15 s	*Enterococcus faecalis* (ATCC 29212)	1.1	1.16×10^5^	CFU/mL	(50)
	72°C, 5 s	*Enterococcus faecalis* (ATCC 29212)	3.8	1.16×10^5^	CFU/mL	(50)
	72°C, 15 s	*Enterococcus faecalis* (ATCC 29212)	3.8	1.16×10^5^	CFU/mL	(50)
	87°C, 2 s	*Enterococcus faecalis* (ATCC 29212)	>5.1	1.16×10^5^	CFU/mL	(50)
	87°C, 5 s	*Enterococcus faecalis* (ATCC 29212)	>5.1	1.16×10^5^	CFU/mL	(50)
	62°C, 5 s	Enterococcus species*	0.92	10^3^	CFU/mL	(48)
	71°C, 18.9 s, 5.9 mL/min	*Escherichia coli* (ATCC 25922)	>5.15	1.40×10^6^	CFU/mL	(57)
	71°C, 9 s, 12.3 mL/min	*Escherichia coli* (ATCC 25922)	>5.15	1.40×10^6^	CFU/mL	(57)
	71°C, 5.75 s, 18.9 mL/min	*Escherichia coli* (ATCC 25922)	>5.15	1.40×10^6^	CFU/mL	(57)
	72°C, 16 s	*Escherichia coli* (CIVO.B.0505)	>32	2.10×10^8^	CFU/mL	(55)
	72°C, 16 s	Hepatitis A Virus (HAV)	2	2.45×10^7^	TCID_50_	(55)
	72°C, 16 s	Human Immunodeficiency Virus (HIV)	>7.27	2.24×10^8^	TCID_50_	(55)
	62°C, 15 s	*Klebsiella pneumoniae* (ATCC 700603)	4.5	5.70×10^4^	CFU/mL	(50)
	72°C, 5 s	*Klebsiella pneumoniae* (ATCC 700603)	>4.8	5.70×10^4^	CFU/mL	(50)
	72°C, 15 s	*Klebsiella pneumoniae* (ATCC 700603)	>4.8	5.70×10^4^	CFU/mL	(50)
	87°C, 2 s	*Klebsiella pneumoniae* (ATCC 700603)	>4.8	5.70×10^4^	CFU/mL	(50)
	62°C, 15 s	*Klebsiella pneumoniae* (Kpn 01605)	1.1	3.60×10^4^	CFU/mL	(50)
	72°C, 5 s	*Klebsiella pneumoniae* (Kpn 01605)	>4.6	3.60×10^4^	CFU/mL	(50)
	72°C, 15 s	*Klebsiella pneumoniae* (Kpn 01605)	>4.6	3.60×10^4^	CFU/mL	(50)
	72°C, 16 s	Porcine Parvovirus (PPV)	0.5	3.80×10^8^	TCID_50_	(55)
	62°C, 5 s	*Pseudomonas aeruginosa* (ATCC 27853)	1.1	10^5^	CFU/mL	(50)
	62°C, 15 s	*Pseudomonas aeruginosa* (ATCC 27853)	3.7	10^5^	CFU/mL	(50)
	72°C, 5 s	*Pseudomonas aeruginosa* (ATCC 27853)	5	10^5^	CFU/mL	(50)
	87°C, 5 s	*Pseudomonas aeruginosa* (ATCC 27853)	5	10^5^	CFU/mL	(50)
	72°C, 16 s	Pseudorabies Virus (PRV)	>7.68	4.37×10^8^	TCID_50_	(55)
	62°C, 5 s	*Serratia marcescens* (Smarc 00697)	3.1	1.04×10^5^	CFU/mL	(50)
	62°C, 15 s	*Serratia marcescens* (Smarc 00697)	>4.9	1.04×10^5^	CFU/mL	(50)
	72°C, 5 s	*Serratia marcescens* (Smarc 00697)	>4.9	1.04×10^5^	CFU/mL	(50)
	87°C, 5 s	*Serratia marcescens* (Smarc 00697)	>4.9	1.04×10^5^	CFU/mL	(50)
	71°C, 18.9 s, 5.9 mL/min	*Staphylococcus aureus* (ATCC 25923)	>6.07	1.20×10^7^	CFU/mL	(57)
	71°C, 9 s, 12.3 mL/min	*Staphylococcus aureus* (ATCC 25923)	>6.07	1.20×10^7^	CFU/mL	(57)
	71°C, 5.75 s, 18.9 mL/min	*Staphylococcus aureus* (ATCC 25923)	>6.07	1.20×10^7^	CFU/mL	(57)
	72°C, 15 s	*Staphylococcus aureus* (ATCC 33862)	4.48	3.00×10^6^	CFU/mL	(56)
	62°C, 5 s	*Staphylococcus aureus* (ATCC 6538)	2.4	7.90×10^4^	CFU/mL	(50)
	72°C, 5 s	*Staphylococcus aureus* (ATCC 6538)	3.3	7.90×10^4^	CFU/mL	(50)
	62°C, 15 s	*Staphylococcus aureus* (ATCC 6538)	3.6	7.90×10^4^	CFU/mL	(50)
	72°C, 15 s	*Staphylococcus aureus* (ATCC 6538)	3.9	7.90×10^4^	CFU/mL	(50)
	87°C, 2 s	*Staphylococcus aureus* (ATCC 6538)	>4.9	7.90×10^4^	CFU/mL	(50)
	87°C, 5 s	*Staphylococcus aureus* (ATCC 6538)	>4.9	7.90×10^4^	CFU/mL	(50)
	72°C, 16 s	*Staphylococcus aureus* (NCCB70054/CIVO.B. 1,245)	15	2.50×10^7^	CFU/mL	(55)
	72°C, 16 s	*Streptococcus agalactiae* (CIVO.B.0062)	>26	3.80×10^6^	CFU/mL	(55)

Microorganisms marked with * were evaluated as native on HBM; the others were inoculated. HoP, Holder Pasteurization; HTST, High-Temperature Short-Time Pasteurization. ^A^CFU, Colony Forming Unit; ^B^PFU, Plaque Forming Unit; ^C^IEA, Immediate Early Antigen; ^D^FFU, Focus Forming Unit; ^E^TCID_50_, 50% of the Tissue Culture Infectivity Dose.

**Table 4:** Effect of thermal processes applied for breast milk preservation on bioactive and nutritional components.

Applied treatment	Process conditions	Bioactive or nutritional components	Initial content	Percentage of retention	Reference
HoP	62.5°C, 30 min	Adiponectin	–	67.21	(58)
	62.5°C, 30 min. After heating: placed in water at 4°C for 1 h	Ascorbic acid	14 ± 11.2 mg/L	38.57	(59)
	62.5°C, 30 min	Bile Salt Stimulated Lipase (BSSL) (activity)	145 ± 22 μmol/mL/min	0.06	(56)
	63°C, 30 min. After heating: ice bath to quickly cool the milk to 4°C	Bile Salt Stimulated Lipase (BSSL) (activity)	9.4 U/mL	0.30	(60)
	62.5°C, 30 min	Bile Salt Stimulated Lipase (BSSL) (activity)	–	1.42 ± 0.68	(61)
	63 ± 0.5°C, 30 min. After heating: cooled with water at 20°C	Bile Salt Stimulated Lipase (BSSL) (concentration)	1804.5 ± 581.7 U/L	0.40	(48)
	62.5°C, 30 min. After heating: placed in water at 4°C for 1 h	Bile Salt Stimulated Lipase (BSSL) (concentration)	–	<1	(59)
	62.5°C, 30 min. After heating: placed in water at 4°C for 1 h	Carbohydrate	69 ± 3 g/L	100	(59)
	63°C, 30 min. After heating: 4°C	Carbohydrate	8.1 (8.0, 8.2) g/dL	101.23	(49)
	62.5°C, 30 min	Catalase (activity)	17.0 ± 1.56 nmol/ min mL	~43	(52)
	64°C, 30 min. After heating: 4°C	Crude protein	1.3 (0.9, 1.5) g/dL	100	(49)
	62.5°C, 30 min. After heating: placed in water at 4°C for 1 h	Crude protein	10 ± 2 g/L	110	(59)
	62.5°C, 30 min. After heating: submerged for 60 min in an ice-cold bath	Cytokine IL-8 (concentration)	119.0 (106.1–123.19) pg./mL	166.05	(62)
	62.5°C, 30 min. After heating: submerged for 60 min in an ice-cold bath	Cytokine IP-10 (concentration)	30.9 (30.3–35.4) pg./mL	115.21	(62)
	62.5°C, 30 min. After heating: submerged for 60 min in an ice-cold bath	Cytokine MCP-1 (concentration)	524.2 (410.9–688.0) pg./mL	82.88	(62)
	62.5°C, 30 min. After heating: submerged for 60 min in an ice-cold bath	Cytokine MIG (concentration)	25.7 (19.4–29.3) pg./mL	114	(62)
	62.5°C, 30 min. After heating: submerged for 60 min in an ice-cold bath	Cytokine TGF- β2 (concentration)	844.7 (671.9–956.8) pg./mL	83.26	(62)
	62.5°C, 30 min. After heating: placed in water at 4°C for 1 h	Energy	616 ± 72kcal/L	99.51	(59)
	65°C, 30 min. After heating: 4°C	Energy	67 (63, 64) Kcal/dL	100	(49)
	66°C, 30 min. After heating: 4°C	Fat	3.3 (2.8, 3.8) g/dL	93.93	(49)
	62.5°C, 30 min. After heating: placed in water at 4°C for 1 h	Fat	31 ± 8 g/L	100	(59)
	62.5°C, 30 min. After heating: placed in water at 4°C for 1 h	Folate (concentration)	191 ± 83 nmol/L	72.77	(59)
	62.5°C, 30 min	Hepatocyte growth factor (HGF)	–	11.28	(58)
	62.5°C, 30 min. After heating: cooled in ice slurry	Immunoglobulin A (IgA) (activity)	0.900 mg/mL	51.20	(63)
	62.5°C, 30 min After heating: cooled immediately in an ice water bath	Immunoglobulin A (IgA) (concentration)	–	49	(65)
	62.7–64.8°C, 42 min	Immunoglobulin A (IgA) (concentration)	–	53.70	(66)
	62.6–62.9°C, 31 min	Immunoglobulin A (IgA) (concentration)	–	71.30	(66)
	62.5°C, 30 min. After heating: rapidly cooled in an ice bath	Immunoglobulin A (IgA) (concentration)	338.45 ± 27.03 μg/mL	72.05	(67)
	63 ± 0.5°C, 30 min. After heating: cooled with water at 20°C	Immunoglobulin A (IgA) (concentration)	51.7 ± 28.3 mg/dL	83	(48)
	62.5°C, 30 min	Immunoglobulin A (IgA) (concentration)	–	48.5 ± 6.13	(61)
	62.5°C, 30 min	Immunoglobulin G (IgG) (concentration)	–	50.96	(58)
	62.5°C, 30 min. After heating: submerged for 60 min in an ice-cold bath	Immunoglobulin G (IgG) (concentration)	21.2 μg/mL	67	(62)
	62.5°C, 30 min	Insulin	–	67.60	(58)
	62.5°C, 30 min. After heating: submerged for 60 min in an ice-cold bath	Lactoferrin (concentration)	2.4 mg/mL	15	(62)
	62.7–64.8°C, 42 min	Lactoferrin (concentration)	–	16.00	(66)
	62.5°C, 30 min. After heating: cooled immediately in an ice water bath	Lactoferrin (concentration)	–	20	(65)
	63 ± 0.5°C, 30 min. After heating: cooled with water at 20°C	Lactoferrin (concentration)	10.7 ± 15.1 mg/mL	20	(48)
	62.6–62.9°C, 31 min	Lactoferrin (concentration)	–	21.60	(66)
	62.5°C, 30 min	Lactoferrin (concentration)	–	39.69	(58)
	67°C, 30 min. After heating: 4°C	Lactoferrin (concentration)	–	65	(49)
	62.5°C, 30 min	Lactoferrin (concentration)	–	20.0 ± 4.34	(61)
	62.5°C, 30 min. After heating: immediately cool down in an ice bath until the temperature reached 4°C.	Lactoferrin (concentration)	3.00 ± 0.83 g/L	9 ± 4	(68)
	62.5°C, 30 min. After heating: cooled immediately in an ice water bath	Lactoperoxidase (LPO) (activity)	–	6	(65)
	62.5°C, 30 min	Lactoperoxidase (LPO) (activity)	–	<20	(61)
	62.5°C, 30 min	Leptin	–	22.14	(58)
	62.5°C, 30 min. After heating: submerged for 60 min in an ice-cold bath	Lipase (concentration)	–	47	(62)
	62.5°C, 30 min	Lipid peroxidase	26.7 ± 1.74 nM/mL	76	(52)
	62.5°C, 30 min	Lipoprotein lipase (LPL) (activity)	–	14.2 ± 2.69	(61)
	62.5°C, 30 min. After heating: cooled immediately in an ice water bath	Lysozyme (activity)	–	35	(65)
	62.5°C, 30 min	Lysozyme (activity)	7,969 ± 1,394 U/mL	54	(69)
	62.5°C, 30 min. After heating: cooled in ice slurry	Lysozyme (activity)	–	60.50	(63)
	62.5°C, 30 min. After heating: submerged for 60 min in an ice-cold bath	Lysozyme (activity)	–	63	(62)
	62.5°C, 30 min	Lysozyme (activity)	50.2 ± 0.2 U/μL	104.18	(56)
	62.5°C, 30 min	Lysozyme (activity)	–	35.2 ± 2.95	(61)
	63 ± 0.5°C, 30 min. After heating: cooled with water at 20°C	Lysozyme (concentration)	59.5 ± 37.9 μg/mL	65	(48)
	62.7–64.8°C, 42 min	Lysozyme (concentration)	–	74.40	(66)
	62.6–62.9°C, 31 min	Lysozyme (concentration)	–	84.20	(66)
	62.5°C, 30 min	Lysozyme (concentration)	140.8 ± 9.46 μg/mL	~53	(52)
	62.5°C, 30 min. After heating: immediately cool down in an ice bath until the temperature reaches 4°C.	Lysozyme (concentration)	0.024 ± 0.015 g/L	41 ± 14	(68)
	62.5°C, 30 min	Secretory IgA (sIgA) (activity)	1.04 ± 0.09 mg active sIgA/mL	87	(69)
	62.5°C, 30 min	Secretory IgA (sIgA) (concentration)	–	46.3 ± 13.2	(56)
	62.5°C, 30 min. After heating: immediately cool down in an ice bath until the temperature reaches 4°C.	Secretory IgA (sIgA) (concentration)	3.45 ± 0.70 g/L	49 ± 3	(68)
	62.5°C, 30 min	Thiobarbituric acid reactive substances (TBARS)	35.8 ± 7.78 μg MDA/ 100 mL	~108.6	(52)
	62.5°C, 30 min	Total antioxidant capacity (TAC)	40.5 ± 5.44 mg TE/100 mL	95.80	(52)
	62.5°C, 30 min. After heating: placed in water at 4°C for 1 h	Total vitamin C	15 ± 12 mg/L	36	(59)
	68°C, 30 min. After heating: 4°C	True Protein	1.0 (0.7, 1.2) g/dL	100	(49)
	62.5°C, 30 min	Vitamin C	53.1 ± 5.29 mg/L	~60	(52)
	62.5°C, 30 min	Xanthine oxidase (XO) (activity)	–	<20	(61)
HTST	72°C, 15 s	Bile Salt Stimulated Lipase (BSSL) (activity)	145 ± 22 μmol/mL/min	0.17	(56)
	62°C, 5 s. After heating: rapidly cooled with water at 14°C	Bile Salt Stimulated Lipase (BSSL) (concentration)	1804.5 ± 581.7 U/L	0.80	(48)
	71°C, 9 s, 12.3 mL/min	Immunoglobulin A (IgA) (concentration)	–	74	(57)
	71°C, 5.8 s, 18.9 mL/min	Immunoglobulin A (IgA) (concentration)	–	82.80	(57)
	62°C, 5 s. After heating: rapidly cooled with water at 14°C	Immunoglobulin A (IgA) (concentration)	51.6 ± 28.3 mg/dL	95	(48)
	72.8°C, 9 s	Immunoglobulin G (IgG) (concentration)	–	57.30	(57)
	71°C, 9 s, 12.3 mL/min	Immunoglobulin G (IgG) (concentration)	–	75	(57)
	71°C, 5.8 s, 18.9 mL/min	Immunoglobulin G (IgG) (concentration)	–	79.10	(57)
	72.8°C, 9 s	Immunoglobulin M (IgM) (concentration)	–	52.20	(57)
	71°C, 9 s, 12.3 mL/min	Immunoglobulin M (IgM) (concentration)	–	68	(57)
	71°C, 5.8 s, 18.9 mL/min	Immunoglobulin M (IgM) (concentration)	–	72.06	(57)
	62°C, 5 s. After heating: rapidly cooled with water at 14°C	Lactoferrin (concentration)	10.7 ± 15.1 mg/mL	32	(48)
	72°C, 15 s	Lysozyme (activity)	50.2 ± 0.2 U/μL	97.21	(56)
	62°C, 5 s. After heating: rapidly cooled with water at 14°C	Lysozyme (concentration)	59.5 ± 37.9 μg/mL	72	(48)
	72°C, 15 s	Secretory IgA (sIgA) (concentration)	–	78.9 ± 2.4	(56)

HoP, Holder Pasteurization; HTST, High-Temperature Short-Time Pasteurization.

**Table 5:** Effect of non-thermal processes applied for pathogen reduction on breast milk.

Applied treatment	Process conditions	Microorganism or pathogen	Log_10_ reductions	Initial load	Units^A-D^	Reference
HPP	593.96 MPa, 233 s, 24.8°C	*Bacillus cereus* (vegetative)	6.93	10^7^	CFU/mL	(47)
	350 MPa, 5 min, 4 cycles, 38°C	*Bacillus cereus* spores (ATCC 14579)	4.9	7.94×10^4^	CFU/mL	(70)
	350 MPa, 8 min, 16.5°C	Cytomegalovirus (CMV)	>0.9	3.16×10^3^	PFU/mL	(49)
	350 MPa, 10 min, 16.5°C	Cytomegalovirus (CMV)	>0.9	3.16×10^3^	PFU/mL	(49)
	500 MPa, 8 min, 18.8°C	Cytomegalovirus (CMV)	>0.9	3.16×10^3^	PFU/mL	(49)
	500 MPa, 10 min, 18.8°C	Cytomegalovirus (CMV)	>0.9	3.16×10^3^	PFU/mL	(49)
	600 MPa, 8 min, 21.5°C	Cytomegalovirus (CMV)	>0.9	3.16×10^3^	PFU/mL	(49)
	600 MPa, 10 min, 21.5°C	Cytomegalovirus (CMV)	>0.9	3.16×10^3^	PFU/mL	(49)
	600 MPa, 5 min, 20°C	Enveloped hepatitis E virus (eHEV)	1.6	3.16×10^4^	FFU/mL	(51)
	350 MPa, 5 min, 4 cycles, 38°C	Enveloped hepatitis E virus (eHEV)	0	3.16×10^4^	FFU/mL	(51)
	400 MPa, 5 min, 12°C	Enterobacteriaceae*	0.48	3.00×10^1^	CFU/mL	(67)
	500 MPa, 5 min, 12°C	Enterobacteriaceae*	0.48	3.00×10^1^	CFU/mL	(67)
	600 MPa, 5 min, 12°C	Enterobacteriaceae*	0.48	3.00×10^1^	CFU/mL	(67)
	400 MPa, 30 min, 21 to 31°C	*Escherichia coli* (ATCC 25922)	6	10^8^	CFU/mL	(53)
	350 MPa, 5 min, 4 cycles, 38°C	HCoV-229E	1.4	10^6^	TCID_50_/mL	(51)
	600 MPa, 5 min, 20°C	HCoV-229E	1	10^6^	TCID_50_/mL	(51)
	350 MPa, 8 min, 16.5°C	*Hepatitis A virus* (HAV)	>4	5.01×10^5^		(49)
	350 MPa, 10 min, 16.5°C	*Hepatitis A virus* (HAV)	>4	5.01×10^5^		(49)
	500 MPa, 8 min, 18.8°C	*Hepatitis A virus* (HAV)	>4	5.01×10^5^		(49)
	500 MPa, 10 min, 18.8°C	*Hepatitis A virus* (HAV)	>4	5.01×10^5^		(49)
	600 MPa, 8 min, 21.5°C	*Hepatitis A virus* (HAV)	>4	5.01×10^5^		(49)
	600 MPa, 10 min, 21.5°C	*Hepatitis A virus* (HAV)	>4	5.01×10^5^		(49)
	400 MPa, 2 min, 21 to 31°C	*Listeria monocytogenes* (ATCC 19115)	8	10^8^	CFU/mL	(53)
	600 MPa, 5 min, 20°C	Non-enveloped hepatitis E virus (neHEV)	2.7	2.51×10^6^	FFU/mL	(51)
	350 MPa, 5 min, 4 cycles, 38°C	Non-enveloped hepatitis E virus (neHEV)	0.3	2.51×10^6^	FFU/mL	(51)
	400 MPa, 30 min, 21 to 31°C	*Staphylococcus aureus* (ATCC 25923)	8	10^8^	CFU/mL	(53)
	350 MPa, 5 min, 4 cycles, 38°C	*Staphylococcus aureus* (ATCC 6538)	5.7	5.01×10^5^	CFU/mL	(70)
	400 MPa, 30 min, 21 to 31°C	*Staphylococcus aureus* (ATCC 6538)	6	10^8^	CFU/mL	(53)
	500 MPa, 10 min, 50°C	*Staphylococcus aureus* (ATCC 6538)	6.6	10^8^	CFU/mL	(71)
	500 MPa, 15 min, 50°C	*Staphylococcus aureus* (ATCC 6538)	8	10^8^	CFU/mL	(71)
	500 MPa, 15 min, 4°C	*Staphylococcus aureus* (ATCC 6538)	~5	10^8^	CFU/mL	(71)
	500 MPa, 15 min, 20°C	*Staphylococcus aureus* (ATCC 6538)	~5	10^8^	CFU/mL	(71)
	593.96 MPa, 233 s, 24.8°C	*Staphylococcus aureus* sub. Aureus	5.81	10^7^	CFU/mL	(47)
	400 MPa, 4 min, 21 to 31°C	*Streptococcus agalactiae* (ATCC 12927)	8	10^8^	CFU/mL	(53)
	400 MPa, 5 min, 12°C	Total bacteria population*	3.46	2.90×10^4^	CFU/mL	(67)
	500 MPa, 5 min, 12°C	Total bacteria population*	3.46	2.90×10^4^	CFU/mL	(67)
	600 MPa, 5 min, 12°C	Total bacteria population*	3.46	2.90×10^4^	CFU/mL	(67)
	500 MPa, 10 min, 20°C	Total microbial count*	4.88	7.50×10^4^		(71)
UV radiation	1.1 W, 253.7 nm, 8.3 min, 105 g/L of total solid	*Bacillus cereus* (ATCC 10702)	5	10^5^	CFU/mL	(68)
	1.1 W, 253.7 nm, 14.8 min, 125 g/L of total solid	*Bacillus cereus* (ATCC 10702)	5	10^5^	CFU/mL	(68)
	1.1 W, 253.7 nm, 26.5 min, 145 g/L of total solid	*Bacillus cereus* (ATCC 10702)	5	10^5^	CFU/mL	(68)
	254 nm, 400 rpm, 2,750 J/L	*Bacillus subtilis spores* (NRRL B-354, 356)	2.75	10^8^	CFU/mL	(60)
	254 nm, 400 rpm, 550 J/L	*Bacillus subtilis spores* (NRRL B-354, 356)	<1.0	10^8^	CFU/mL	(60)
	254 nm, 400 rpm, 8,250 J/L	*Bacillus subtilis spores* (NRRL B-354, 356)	>5	10^8^	CFU/mL	(60)
	254 nm, 400 rpm, 33,000 J/L	*Bacillus subtilis spores* (NRRL B-354, 356)	Below detection limit	10^8^	CFU/mL	(60)
	254 nm, 400 rpm, 550 J/L	*Cronobacter sakazakii* (ATCC BAA-894)	<1.0	10^8^	CFU/mL	(60)
	254 nm, 400 rpm, 8,250 J/L	*Cronobacter sakazakii* (ATCC BAA-894)	>5	10^8^	CFU/mL	(60)
	254 nm, 400 rpm, 2,750 J/L	*Cronobacter sakazakii* (ATCC BAA-894)	3.64 to 4.82	10^8^	CFU/mL	(60)
	254 nm, 400 rpm, 33,000 J/L	*Cronobacter sakazakii* (ATCC BAA-894)	Below detection limit	10^8^	CFU/mL	(60)
	1.1 W, 253.7 nm, 8.3 min, 105 g/L of total solid	*Enterobacter cloacae* (ATCC 27508)	5	10^5^	CFU/mL	(68)
	1.1 W, 253.7 nm, 14.8 min, 125 g/L of total solid	*Enterobacter cloacae* (ATCC 27508)	5	10^5^	CFU/mL	(68)
	1.1 W, 253.7 nm, 26.5 min, 145 g/L of total solid	*Enterobacter cloacae* (ATCC 27508)	5	10^5^	CFU/mL	(68)
	254 nm, 40 min, 740 J/L	*Enterococcus faecalis* (PCM 896)	2.9	10^5^	CFU/mL	(52)
	254 nm, 40 min, 740 J/L	*Enterococcus faecium* (ATCC 6057)	3.95	10^5^	CFU/mL	(52)
	254 nm, 400 rpm, 550 J/L	*Enterococcus faecium* (ATCC 8459)	<1.0	10^8^	CFU/mL	(60)
	254 nm, 400 rpm, 8,250 J/L	*Enterococcus faecium* (ATCC 8459)	>5	10^8^	CFU/mL	(60)
	254 nm, 400 rpm, 2,750 J/L	*Enterococcus faecium* (ATCC 8459)	3.64 to 4.82	10^8^	CFU/mL	(60)
	254 nm, 400 rpm, 33,000 J/L	*Enterococcus faecium* (ATCC 8459)	Below detection limit	10^8^	CFU/mL	(60)
	254 nm, 400 J/L	*Escherichia coli* (K-12)	Below detection limit	10^5^	CFU/mL	(52)
	254 nm, 700 J/L	*Escherichia coli* (K-12)	Below detection limit	10^5^	CFU/mL	(52)
	1.1 W, 253.7 nm, 8.3 min, 105 g/L of total solid	*Escherichia coli* K 12 (ATCC1498)	5	10^5^	CFU/mL	(68)
	1.1 W, 253.7 nm, 14.8 min, 125 g/L of total solid	*Escherichia coli* K 12 (ATCC1498)	5	10^5^	CFU/mL	(68)
	1.1 W, 253.7 nm, 26.5 min, 145 g/L of total solid	*Escherichia coli* K 12 (ATCC1498)	5	10^5^	CFU/mL	(68)
	254 nm, 400 rpm, 550 J/L	*Listeria monocytogenes* (ScottA, OSY-428, Ohio, California, ATCC 19115)	<1.0	10^8^	CFU/mL	(60)
	254 nm, 400 rpm, 8,250 J/L	*Listeria monocytogenes* (ScottA, OSY-428, Ohio, California, ATCC 19115)	>5	10^8^	CFU/mL	(60)
	254 nm, 400 rpm, 2,750 J/L	*Listeria monocytogenes* (ScottA, OSY-428, Ohio, California, ATCC 19115)	3.64 to 4.82	10^8^	CFU/mL	(60)
	254 nm, 400 rpm, 33,000 J/L	*Listeria monocytogenes* (ScottA, OSY-428, Ohio, California, ATCC 19115)	Below detection limit	10^8^	CFU/mL	(60)
	254 nm, 400 rpm, 2,750 J/L	*Paenibacillus macerans spores* (NRRL B-14029)	2.75	10^8^	CFU/mL	(60)
	254 nm, 400 rpm, 550 J/L	*Paenibacillus macerans spores* (NRRL B-14029)	<1.0	10^8^	CFU/mL	(60)
	254 nm, 400 rpm, 8,250 J/L	*Paenibacillus macerans spores* (NRRL B-14029)	>5	10^8^	CFU/mL	(60)
	254 nm, 400 rpm, 33,000 J/L	*Paenibacillus macerans spores* (NRRL B-14029)	Below detection limit	10^8^	CFU/mL	(60)
	254 nm, 400 rpm, 2,750 J/L	*Paenibacillus polymyxa spores* (NRRL B-510)	2.75	10^8^	CFU/mL	(60)
	254 nm, 400 rpm, 550 J/L	*Paenibacillus polymyxa spores* (NRRL B-510)	<1.0	10^8^	CFU/mL	(60)
	254 nm, 400 rpm, 8,250 J/L	*Paenibacillus polymyxa spores* (NRRL B-510)	>5	10^8^	CFU/mL	(60)
	254 nm, 400 rpm, 33,000 J/L	*Paenibacillus polymyxa spores* (NRRL B-510)	Below detection limit	10^8^	CFU/mL	(60)
	254 nm, 400 rpm, 550 J/L	*Staphylococcus aureus* (138-CPS and 146-CPS)	<1.0	10^8^	CFU/mL	(60)
	254 nm, 400 rpm, 8,250 J/L	*Staphylococcus aureus* (138-CPS and 146-CPS)	>5	10^8^	CFU/mL	(60)
	254 nm, 400 rpm, 2,750 J/L	*Staphylococcus aureus* (138-CPS and 146-CPS)	3.64 to 4.82	10^8^	CFU/mL	(60)
	254 nm, 400 rpm, 33,000 J/L	*Staphylococcus aureus* (138-CPS and 146-CPS)	Below detection limit	10^8^	CFU/mL	(60)
	1.1 W, 253.7 nm, 8.3 min, 105 g/L of total solid	*Staphylococcus aureus* (ATCC 6538)	5	10^5^	CFU/mL	(68)
	1.1 W, 253.7 nm, 14.8 min, 125 g/L of total solid	*Staphylococcus aureus* (ATCC 6538)	5	10^5^	CFU/mL	(68)
	1.1 W, 253.7 nm, 26.5 min, 145 g/L of total solid	*Staphylococcus aureus* (ATCC 6538)	5	10^5^	CFU/mL	(68)
	254 nm, 400 J/L	*Staphylococcus aureus* (PCM 2054)	Below detection limit	10^5^	CFU/mL	(52)
	254 nm, 700 J/L	*Staphylococcus aureus* (PCM 2054)	Below detection limit	10^5^	CFU/mL	(52)
	1.1 W, 253.7 nm, 8.3 min, 105 g/L of total solid	*Staphylococcus epidermidis* (ATCC 12228)	5	10^5^	CFU/mL	(68)
	1.1 W, 253.7 nm, 14.8 min, 125 g/L of total solid	*Staphylococcus epidermidis* (ATCC 12228)	5	10^5^	CFU/mL	(68)
	1.1 W, 253.7 nm, 26.5 min, 145 g/L of total solid	*Staphylococcus epidermidis* (ATCC 12228)	5	10^5^	CFU/mL	(68)
PEF	7 kV, 1,500 pulses, 10 Hz, 30 J	Endogenous bacteria*	0.42	3.16×10^7^	CFU/mL	(65)
	7 kV, 1,500 pulses, 10 Hz, 37.5 J	Endogenous bacteria*	0.46	3.16×10^7^	CFU/mL	(65)
	7 kV, 3,750 pulses, 20 Hz, 93.75 J	Endogenous bacteria*	0.5	3.16×10^7^	CFU/mL	(65)
	7 kV, 1,500 pulses, 50 Hz, 37.5 J	Endogenous bacteria*	0.5	3.16×10^7^	CFU/mL	(65)
	7 kV, 6,000 pulses, 20 Hz, 150 J	Endogenous bacteria*	0.57	3.16×10^7^	CFU/mL	(65)
	7 kV, 6,000 pulses, 50 Hz, 180 J	Endogenous bacteria*	0.58	3.16×10^7^	CFU/mL	(65)
	15 kV, 1,500 pulses, 50 Hz, 210 J	Endogenous bacteria*	0.59	3.16×10^7^	CFU/mL	(65)
	7 kV, 1,500 pulses, 20 Hz, 30 J	Endogenous bacteria*	0.59	3.16×10^7^	CFU/mL	(65)
	11 kV, 6,000 pulses, 10 Hz, 360 J	Endogenous bacteria*	0.61	3.16×10^7^	CFU/mL	(65)
	11 kV, 3,750 pulses, 50 Hz, 225 J	Endogenous bacteria*	0.67	3.16×10^7^	CFU/mL	(65)
	11 kV, 1,500 pulses, 20 Hz, 85.5 J	Endogenous bacteria*	0.7	3.16×10^7^	CFU/mL	(65)
	7 kV, 6,000 pulses, 10 Hz, 120 J	Endogenous bacteria*	0.7	3.16×10^7^	CFU/mL	(65)
	15 kV, 1,500 Pulses, 10 Hz, 165 J	Endogenous bacteria*	0.8	3.16×10^7^	CFU/mL	(65)
	11 kV, 6,000 pulses, 20 Hz, 360 J	Endogenous bacteria*	0.82	3.16×10^7^	CFU/mL	(65)
	15 kV, 1,500 pulses, 20 Hz, 165 J	Endogenous bacteria*	1.37	3.16×10^7^	CFU/mL	(65)
	15 kV, 3,750 pulses, 10 Hz, 450 J	Endogenous bacteria*	2.27	3.16×10^7^	CFU/mL	(65)
	15 kV, 6,000 pulses, 10 Hz, 660 J	Endogenous bacteria*	2.3	3.16×10^7^	CFU/mL	(65)
	15 kV, 3,750 pulses, 20 Hz, 450 J	Endogenous bacteria*	3.19	3.16×10^7^	CFU/mL	(65)
	15 kV, 6,000 pulses, 20 Hz, 780 J	Endogenous bacteria*	3.94	3.16×10^7^	CFU/mL	(65)
	15 kV, 6,000 pulses, 50 Hz, 720 J	Endogenous bacteria*	4.67	3.16×10^7^	CFU/mL	(65)

Microorganisms marked with * were evaluated as native on HBM, the others were inoculated. HPP, High-Pressure Processing; PEF, Pulsed Electric Fields. ^A^CFU, Colony Forming Unit; ^B^PFU, Plaque Forming Unit; ^C^FFU, Focus Forming Unit; ^D^TCID_50_, 50% of the Tissue Culture Infectivity Dose.

**Table 6:** Effect of non-thermal processes applied for breast milk preservation on bioactive and nutritional components.

Applied treatment	Process conditions	Bioactive or nutritional components	Initial content	Percentage of retention	Reference
HPP	600 MPa, 10 min, 19–21°C	Adiponectin	–	2.01	(58)
	200 MPa, 10 min; interval, 10 min; 600 MPa, 10 min, 19–21°C	Adiponectin	–	4.09	(58)
	100 MPa, 10 min; interval 10 min; 600 MPa, 10 min, 19–21°C	Adiponectin	–	10.73	(58)
	200 MPa, 10 min; interval 10 min; 400 MPa, 10 min, 19–21°C	Adiponectin	–	38.55	(58)
	500 MPa, 8 min, 4°C	Ascorbic acid	14 ± 11.2 mg/L	23.57	(59)
	550 MPa, 5 min	Bile Salt Stimulated Lipase (BSSL) (activity)	9.4 U/mL	61.60	(60)
	500 MPa, 8 min, 4°C	Bile Salt Stimulated Lipase (BSSL) (activity)	–	100	(59)
	400 MPa, 5 min, 25°C	Bile Salt Stimulated Lipase (BSSL) (activity)	–	110 ± 6.53	(61)
	500 MPa, 8 min, 4°C	Carbohydrate	69 ± 3 g/L	97.10	(59)
	350 MPa, 8 min, 16.5°C	Carbohydrate	8.1 (8.0, 8.2) g/dL	100	(49)
	350 MPa, 10 min, 16.5°C	Carbohydrate	8.1 (8.0, 8.2) g/dL	100	(49)
	500 MPa, 8 min, 18.8°C	Carbohydrate	8.1 (8.0, 8.2) g/dL	100	(49)
	500 MPa, 10 min, 18.8°C	Carbohydrate	8.1 (8.0, 8.2) g/dL	100	(49)
	600 MPa, 8 min, 21.5°C	Carbohydrate	8.1 (8.0, 8.2) g/dL	100	(49)
	600 MPa, 10 min, 21.5°C	Carbohydrate	8.1 (8.0, 8.2) g/dL	100	(49)
	350 MPa, 8 min, 16.5°C	Crude protein	1.3 (0.9, 1.5) g/dL	100	(49)
	350 MPa, 10 min, 16.5°C	Crude protein	1.3 (0.9, 1.5) g/dL	100	(49)
	500 MPa, 8 min, 18.8°C	Crude protein	1.3 (0.9, 1.5) g/dL	100	(49)
	500 MPa, 10 min, 18.8°C	Crude protein	1.3 (0.9, 1.5) g/dL	100	(49)
	600 MPa, 8 min, 21.5°C	Crude protein	1.3 (0.9, 1.5) g/dL	100	(49)
	600 MPa, 10 min, 21.5°C	Crude protein	1.3 (0.9, 1.5) g/dL	100	(49)
	425 MPa, 4 cycles, 6 min each, 4°C	Cytokine IL-8 (concentration)	119.0 (106.1–123.1) pg./mL	70.00	(62)
	425 MPa, 4 cycles, 6 min each, 37°C	Cytokine IL-8 (concentration)	119.0 (106.1–123.1) pg./mL	88.32	(62)
	425 MPa, 4 cycles, 6 min each, 4°C	Cytokine IP-10 (concentration)	30.9 (30.3–35.4) pg./mL	60.84	(62)
	425 MPa, 4 cycles, 6 min each, 37°C	Cytokine IP-10 (concentration)	30.9 (30.3–35.4) pg./mL	88.67	(62)
	425 MPa, 4 cycles, 6 min each, 37°C	Cytokine MCP-1 (concentration)	524.2 (410.9–688.0) pg./mL	69.42	(62)
	425 MPa, 4 cycles, 6 min each, 4°C	Cytokine MCP-1 (concentration)	524.2 (410.9–688.0) pg./mL	87.77	(62)
	425 MPa, 4 cycles, 6 min each, 37°C	Cytokine MIG (concentration)	25.7 (19.4–29.3) pg./mL	61.87	(62)
	425 MPa, 4 cycles, 6 min each, 4°C	Cytokine MIG (concentration)	25.7 (19.4–29.3) pg./mL	71.60	(62)
	425 MPa, 4 cycles, 6 min each, 4°C	Cytokine TGF-β2 (concentration)	844.7 (671.9–956.8) pg./mL	71.63	(62)
	425 MPa, 4 cycles, 6 min each, 37°C	Cytokine TGF-β2 (concentration)	844.7 (671.9–956.8) pg./mL	90.81	(62)
	500 MPa, 8 min, 4°C	Energy	616 ± 72 kcal/L	95.94	(59)
	350 MPa, 8 min, 16.5°C	Energy	67 (63, 74) Kcal/dL	97.01	(49)
	350 MPa, 10 min, 16.5°C	Energy	67 (63, 74) Kcal/dL	97.01	(49)
	600 MPa, 10 min, 21.5°C	Energy	67 (63, 74) Kcal/dL	97.01	(49)
	500 MPa, 8 min, 18.8°C	Energy	67 (63, 74) Kcal/dL	98.51	(49)
	500 MPa, 10 min, 18.8°C	Energy	67 (63, 74) Kcal/dL	98.51	(49)
	600 MPa, 8 min, 21.5°C	Energy	67 (63, 74) Kcal/dL	98.51	(49)
	350 MPa, 8 min, 16.5°C	Fat	3.3 (2.8, 3.8) g/dL	87.88	(49)
	350 MPa, 10 min, 16.5°C	Fat	3.3 (2.8, 3.8) g/dL	87.88	(49)
	500 MPa, 8 min, 18.8°C	Fat	3.3 (2.8, 3.8) g/dL	90.91	(49)
	500 MPa, 10 min, 18.8°C	Fat	3.3 (2.8, 3.8) g/dL	90.91	(49)
	600 MPa, 8 min, 21.5°C	Fat	3.3 (2.8, 3.8) g/dL	90.91	(49)
	600 MPa, 10 min, 21.5°C	Fat	3.3 (2.8, 3.8) g/dL	90.91	(49)
	500 MPa, 8 min, 4°C	Fat	31 ± 8 g/L	93.55	(59)
	500 MPa, 8 min, 4°C	Folate (concentration)	191 ± 83 nmol/L	92.67	(59)
	600 MPa, 10 min, 19–21°C	Hepatocyte growth factor (HGF)	–	36.15	(58)
	100 MPa, 10 min; interval 10 min; 600 MPa, 10 min, 19–21°C	Hepatocyte growth factor (HGF)	–	38.81	(58)
	200 MPa, 10 min; interval, 10 min; 600 MPa, 10 min, 19–21°C	Hepatocyte growth factor (HGF)	–	43.02	(58)
	200 MPa, 10 min; interval 10 min; 400 MPa, 10 min, 19–21°C	Hepatocyte growth factor (HGF)	–	97.15	(58)
	400 MPa, 120 min	Immunoglobulin A (IgA) (activity)	0.900 ± 0.035 mg/mL	75.40	(63)
	400 MPa, 90 min	Immunoglobulin A (IgA) (activity)	0.900 ± 0.035 mg/mL	80.60	(63)
	400 MPa, 30 min	Immunoglobulin A (IgA) (activity)	0.900 ± 0.035 mg/mL	85.60	(63)
	400 MPa, 60 min	Immunoglobulin A (IgA) (activity)	0.900 ± 0.035 mg/mL	87.10	(63)
	600 MPa, 5 min, 12°C	Immunoglobulin A (IgA) (concentration)	367.6 ± 38.18 μg/mL	69.31	(67)
	500 MPa, 5 min, 12°C	Immunoglobulin A (IgA) (concentration)	367.6 ± 38.18 μg/mL	87.93	(67)
	400 MPa, 5 min, 12°C	Immunoglobulin A (IgA) (concentration)	367.6 ± 38.18 μg/mL	~100	(67)
	400 MPa, 5 min, 25°C	Immunoglobulin A (IgA) (concentration)	–	97.8 ± 8.74	(61)
	600 MPa, 10 min, 19–21°C	Immunoglobulin G (IgG) (concentration)	–	30.32	(58)
	100 MPa, 10 min; interval 10 min; 600 MPa, 10 min, 19–21°C	Immunoglobulin G (IgG) (concentration)	–	30.84	(58)
	200 MPa, 10 min; interval, 10 min; 600 MPa, 10 min, 19–21°C	Immunoglobulin G (IgG) (concentration)	–	31.54	(58)
	200 MPa, 10 min; interval 10 min; 400 MPa, 10 min, 19–21°C	Immunoglobulin G (IgG) (concentration)	–	82.24	(58)
	100 MPa, 10 min; interval 10 min; 600 MPa, 10 min, 19–21°C	Insulin	–	81.98	(58)
	600 MPa, 10 min, 19–21°C	Insulin	–	88.20	(58)
	200 MPa, 10 min; interval, 10 min; 600 MPa, 10 min, 19–21°C	Insulin	–	90.31	(58)
	200 MPa, 10 min; interval 10 min; 400 MPa, 10 min, 19–21°C	Insulin	–	94.76	(58)
	600 MPa, 10 min, 19–21°C	Lactoferrin (concentration)	–	55.78	(58)
	100 MPa, 10 min; interval 10 min; 600 MPa, 10 min, 19–21°C	Lactoferrin (concentration)	–	57.63	(58)
	200 MPa, 10 min; interval, 10 min; 600 MPa, 10 min, 19–21°C	Lactoferrin (concentration)	–	64.75	(58)
	425 MPa, 4 cycles, 6 min each, 4°C	Lactoferrin (concentration)	2.4 mg/mL	66.67	(62)
	200 MPa, 10 min; interval 10 min; 400 MPa, 10 min, 19–21°C	Lactoferrin (concentration)	–	78.77	(58)
	425 MPa, 4 cycles, 6 min each, 37°C	Lactoferrin (concentration)	2.4 mg/mL	83.33	(62)
	400 MPa, 5 min, 25°C	Lactoferrin (concentration)	–	86.8 ± 10.3	(61)
	400 MPa, 5 min, 25°C	Lactoperoxidase (LPO) (activity)	–	91.4 ± 6.02	(61)
	425 MPa, 4 cycles, 6 min each, 37°C	Lipase (concentration)	–	65	(62)
	400 MPa, 5 min, 25°C	Lipoprotein lipase (LPL) (activity)	–	103 ± 4.76	(61)
	425 MPa, 4 cycles, 6 min each, 4°C	Lysozyme (activity)	–	94	(62)
	400 MPa, 120 min	Lysozyme (activity)	2254.7 ± 207.9 units/mg solid	95.80	(63)
	400 MPa, 60 min	Lysozyme (activity)	2254.7 ± 207.9 units/mg solid	96.30	(63)
	400 MPa, 90 min	Lysozyme (activity)	2254.7 ± 207.9 units/mg solid	96.30	(63)
	425 MPa, 4 cycles, 6 min each, 37°C	Lysozyme (activity)	–	98	(62)
	400 MPa, 30 min	Lysozyme (activity)	2254.7 ± 207.9 units/mg solid	106.90	(63)
	400 MPa, 5 min, 25°C	Lysozyme (activity)	–	119 ± 8.86	(61)
	500 MPa, 8 min, 4°C	Total vitamin C	15 ± 12 mg/L	24.67	(59)
	350 MPa, 8 min, 16.5°C	True protein	1.0 (0.7, 1.2) g/dL	100	(49)
	350 MPa, 10 min, 16.5°C	True protein	1.0 (0.7, 1.2) g/dL	100	(49)
	500 MPa, 8 min, 18.8°C	True protein	1.0 (0.7, 1.2) g/dL	100	(49)
	500 MPa, 10 min, 18.8°C	True protein	1.0 (0.7, 1.2) g/dL	100	(49)
	600 MPa, 8 min, 21.5°C	True protein	1.0 (0.7, 1.2) g/dL	100	(49)
	600 MPa, 10 min, 21.5°C	True protein	1.0 (0.7, 1.2) g/dL	100	(49)
UV radiation	254 nm, 40 min, 740 J/L	Vitamin C	34.5 ± 3.67 mg/L	64.60	(52)
	253.7 nm, 1.1 W, 2084 J/L	Alkaline Phosphatase (ALP) (activity)	0.200 ± 0.050 U/mL	99.50	(72)
	253.7 nm, 1.1 W, 3474 J/L	Alkaline Phosphatase (ALP) (activity)	0.200 ± 0.050 U/mL	99.50	(72)
	253.7 nm, 1.1 W, 4863 J/L	Alkaline Phosphatase (ALP) (activity)	0.200 ± 0.050 U/mL	102.00	(72)
	250 nm, 25 min	Ascorbic acid	14 ± 11.2 mg/L	20.71	(59)
	254 nm, 1.1 W, 33,000 J/L	Bile Salt Stimulated Lipase (BSSL) (activity)	9.4 U/mL	20	(60)
	254 nm, 1.1 W, 16,500 J/L	Bile Salt Stimulated Lipase (BSSL) (activity)	9.4 U/mL	35.50	(60)
	254 nm, 1.1 W, 5,500 J/L	Bile Salt Stimulated Lipase (BSSL) (activity)	9.4 U/mL	69.15	(60)
	254 nm, 1.1 W, 1,100 J/L	Bile Salt Stimulated Lipase (BSSL) (activity)	9.4 U/mL	88.30	(60)
	253.7 nm, 1.1 W, 2084 J/L	Bile Salt Stimulated Lipase (BSSL) (activity)	116.5 ± 36.6 U/mL	96.74	(72)
	253.7 nm, 1.1 W, 3474 J/L	Bile Salt Stimulated Lipase (BSSL) (activity)	116.5 ± 36.6 U/mL	97.51	(72)
	253.7 nm, 1.1 W, 4863 J/L	Bile Salt Stimulated Lipase (BSSL) (activity)	116.5 ± 36.6 U/mL	98.97	(72)
	250 nm, 25 min	Carbohydrate	69 ± 3 g/L	100	(59)
	254 nm, 30 min, 544 J/L	Catalase (activity)	18.4 ± 1.17 nmol/ min mL	85.30	(52)
	254 nm, 5 min, 85 J/L	Catalase (activity)	18.4 ± 1.17 nmol/ min mL	96.80	(52)
	250 nm, 25 min	Crude protein	10 ± 2 g/L	110.00	(59)
	250 nm, 25 min	Energy	616 ± 72 kcal/L	99.03	(59)
	250 nm, 25 min	Fat	31 ± 8 g/L	100	(59)
	250 nm, 25 min	Folate (concentration)	191 ± 83 nmol/L	74.87	(59)
	253.7 nm, 1.1 W, 4683 J/L	Lactoferrin (concentration)	–	87 ± 11	(68)
	253.7 nm, 1.1 W, 3474 J/L	Lactoferrin (concentration)	–	93 ± 10	(68)
	253.7 nm, 1.1 W, 2084 J/L	Lactoferrin (concentration)	–	95 ± 6	(68)
	254 nm, 5 min, 85 J/L	Lipid peroxidase	10.8 ± 1.25 nM/mL	99.10	(52)
	254 nm, 40 min, 740 J/L	Lysozyme (concentration)	152.9 ± 14.89 μg/ mL	59.10	(52)
	254 nm, 30 min, 544 J/L	Lysozyme (concentration)	152.9 ± 14.89 μ/mL	61.00	(52)
	254 nm, 20 min, 355 J/L	Lysozyme (concentration)	152.9 ± 14.89 μg/mL	70.20	(52)
	254 nm, 10 min, 173 J/L	Lysozyme (concentration)	152.9 ± 14.89 μg/mL	91.60	(52)
	254 nm, 5 min, 85 J/L	Lysozyme (concentration)	152.9 ± 14.89 μg/mL	105.80	(52)
	253.7 nm, 1.1 W, 4683 J/L	Lysozyme (concentration)	–	75 ± 9	(68)
	253.7 nm, 1.1 W, 3474 J/L	Lysozyme (concentration)	–	84 ± 10	(68)
	253.7 nm, 1.1 W, 2084 J/L	Lysozyme (concentration)	–	91 ± 7	(68)
	253.7 nm, 1.1 W, 4683 J/L	Secretory IgA (sIgA) (concentration)	–	89 ± 4	(68)
	253.7 nm, 1.1 W, 3474 J/L	Secretory IgA (sIgA) (concentration)	–	94 ± 4	(68)
	253.7 nm, 1.1 W, 2084 J/L	Secretory IgA (sIgA) (concentration)	–	95 ± 5	(68)
	254 nm, 5 min, 85 J/L	Thiobarbituric acid reactive substances (TBARS)	37.1 ± 4.60 μg MDA/ 100 mL	102.70	(52)
	254 nm, 10 min, 173 J/L	Thiobarbituric acid reactive substances (TBARS)	37.1 ± 4.60 μg MDA/ 100 mL	109.20	(52)
	254 nm, 20 min, 355 J/L	Thiobarbituric acid reactive substances (TBARS)	37.1 ± 4.60 μg MDA/ 100 mL	112.40	(52)
	254 nm, 30 min, 544 J/L	Thiobarbituric acid reactive substances (TBARS)	37.1 ± 4.60 μg MDA/ 100 mL	125.30	(52)
	254 nm, 40 min, 740 J/L	Thiobarbituric acid reactive substances (TBARS)	37.1 ± 4.60 μg MDA/ 100 mL	135.30	(52)
	254 nm, 40 min, 740 J/L	Total antioxidant capacity (TAC)	23.1 ± 2.81 mg TE/ 100 mL	100.90	(52)
	254 nm, 30 min, 544 J/L	Total antioxidant capacity (TAC)	23.1 ± 2.81 mg TE/ 100 mL	102.20	(52)
	254 nm, 5 min, 85 J/L	Total antioxidant capacity (TAC)	23.1 ± 2.81 mg TE/ 100 mL	103.90	(52)
	254 nm, 10 min, 173 J/L	Total antioxidant capacity (TAC)	23.1 ± 2.81 mg TE/ 100 mL	106.10	(52)
	254 nm, 20 min, 355 J/L	Total antioxidant capacity (TAC)	23.1 ± 2.81 mg TE/ 100 mL	106.50	(52)
	250 nm, 25 min	Total vitamin C	15 ± 12 mg/L	28.00	(59)
	254 nm, 30 min, 544 J/L	Vitamin C	34.5 ± 3.67 mg/L	73.60	(52)
	254 nm, 20 min, 355 J/L	Vitamin C	34.5 ± 3.67 mg/L	77.10	(52)
	254 nm, 10 min, 173 J/L	Vitamin C	34.5 ± 3.67 mg/L	85.20	(52)
	254 nm, 5 min, 85 J/L	Vitamin C	34.5 ± 3.67 mg/L	94.80	(52)
PEF	15 kV, 6,000 pulses, 50 Hz (PEF-50)	Immunoglobulin A (IgA) (concentration)	–	68	(65)
	15 kV, 6,000 pulses, 20 Hz (PEF-20)	Immunoglobulin A (IgA) (concentration)	–	108	(65)
	15 kV, 6,000 pulses, 50 Hz (PEF-50)	Lactoferrin (concentration)	–	52	(65)
	15 kV, 6,000 pulses, 20 Hz (PEF-20)	Lactoferrin (concentration)	–	74	(65)
	15 kV, 6,000 pulses, 20 Hz (PEF-20)	Lactoperoxidase (LPO) (activity)	–	>60	(65)
	15 kV, 6,000 pulses, 50 Hz (PEF-50)	Lactoperoxidase (LPO) (activity)	–	>60	(65)
	15 kV, 6,000 pulses, 20 Hz (PEF-20)	Lysozyme (activity)	–	76	(65)
	15 kV, 6,000 pulses, 50 Hz (PEF-50)	Lysozyme (activity)	–	80	(65)
	15 kV, 6,000 pulses, 20 Hz (PEF-20)	Xanthine oxidase (activity)	–	100	(65)
	15 kV, 6,000 pulses, 50 Hz (PEF-50)	Xanthine oxidase (activity)	–	100	(65)

HPP, High-Pressure Processing; PEF, Pulsed Electric Fields.

From a microbiological perspective, the most frequently reported microorganisms were *Staphylococcus aureus* (ATCC 6538), *Staphylococcus aureus* (ATCC 25923), Hepatitis A virus (HAV), Cytomegalovirus [in the form of Cytomegalovirus (CMV) and Cytomegalovirus (CMV) (AD169)], *Enterococcus* [in the form of *Enterococcus faecalis* (ATCC 29212), *Enterococcus faecalis* (PCM 896), *Enterococcus faecium* (ATCC 6057), *Enterococcus faecium* (ATCC 8459) and *Enterococcus* spp.], as well as *Escherichia coli* [in the form of *E. coli* (ATCC 25922), *E. coli* (CIVO.B.0505) and *E. coli* (K-12)]. It is hypothesized that the study of these microorganisms is relevant because they are species commonly found in HBM as contaminants from the mother’s epidermis or frequent parasites of the milk.

On the other hand, the nutritional and bioactive compounds in HBM more frequently reported were: lactoferrin concentration, lysozyme concentration, lysozyme activity, immunoglobulin A (IgA), immunoglobulin G (IgG), crude proteins content, carbohydrate content, and the bile salt stimulated lipase (BSSL) activity, which aids in the digestion of lipids by newborn babies (60). Almost all these components have effects in developing crucial characteristics and protecting infants against infection. Also, being predominantly proteins, they are sensitive to heat.

## Thermal processes for breast milk preservation

### Holder Pasteurization (HoP)

Holder Pasteurization (HoP) operates on the principle of heating at a moderate temperature over a sustained period (73). When applied to HBM, the milk is gently raised to about 62.5°C and maintained at that temperature for 30 min. The main aim of HoP is to eradicate harmful microorganisms while minimizing any detrimental impact on the vital nutritional and bioactive elements in breast milk (73); however, its prolonged duration could reduce heat-sensitive components, potentially altering milk’s nutritional composition.

Additionally, while HoP is effective against several pathogens, it might not match the inactivation level achieved by higher-temperature methods. The milk’s initial microbial load is critical in determining HoP effectiveness, emphasizing the need for rigorous quality control measures (74).

Its foundational role in food, biotechnology, and infant care requires a comprehensive understanding of its benefits and challenges. Such insights inform the optimization of HoP and highlight its continued importance in infant nutrition and health (75).

### High-Temperature Short-Time Pasteurization (HTST)

The High-Temperature Short-Time (HTST) method represents a transformative milestone in dairy processing. Introduced in the early 20th century to address the constraints of traditional pasteurization, HTST employs higher temperatures for faster processing. It employs metal plates and hot water to elevate milk temperature quickly, followed by rapid cooling. This method has a crucial role in contemporary food preservation (76).

Central to HTST’s effectiveness is its use of elevated temperatures compared to HoP. Exposing HBM to approximately 72°C for about 15 s swiftly eradicates pathogens without significantly altering its sensory and nutritional attributes.

The technique’s speed and ability to maintain the integrity of HBM are its distinct benefits, positioning it as ideal for large-scale production (77). The short heat exposure better preserves sensitive nutrients and bioactive compounds within HBM, retaining significantly higher levels of immunoglobulins and lactoferrin than traditional pasteurization (78, 79).

However, potential drawbacks include the possibility of protein denaturation, potentially affecting their nutritional quality. While effective against many pathogens, HTST may only comprehensively address some types. The swiftness of HTST might also pose concerns regarding its adaptability to varying microbial challenges (78).

### Effect of thermal processes on breast milk microbiological load

According to Table 3 (47–50, 52, 53), HoP can decrease the load of *Staphylococcus aureus* (ATCC 6538) in at least 4.9 logarithmic (4.9-log_10_) cycles, achieving reductions even more significant than 7-log_10_. For *Staphylococcus aureus* (ATCC 25923), HoP could reduce the microorganism charge by more than 7-log_10_. For *Enterococcus* in the form of *Enterococcus faecalis* (ATCC 29212), *Enterococcus faecalis* (PCM 896), *Enterococcus faecium* (ATCC 6057), and *Enterococcus* spp., this technique has shown a logarithmic reduction from 3.92-log_10_ up to total elimination, being *Enterococcus faecalis* (PCM 896) the most thermoresistant. After HoP, *Escherichia coli* has shown reductions higher than 7-log_10_. In terms of viral inactivation, HoP has shown to be capable of decreasing from >0.9-log_10_ to completely inactivate Cytomegalovirus, while it has achieved a 3.9-log_10_ decrease in Hepatitis A virus (HAV). Among the few studies evaluating HBM processing methods on spore-forming microorganisms, one significant is reference (47), which compares the effect of HPP and HoP on *Bacillus cereus*. The study found that while HoP achieved a 2.62 log CFU/mL reduction in *B. cereus*, HPP was more effective, yielding a 6.93 log CFU/mL reduction. This highlights HPP superior capability in mitigating spore-forming bacteria compared to HoP, which, altogether with HTST (thermal-based treatments) struggle to effectively neutralize bacterial spores. These spores can survive thermal pasteurization and potentially grow at refrigeration temperatures, posing significant food safety concerns, especially in low-acid foods like milk in the context of HBM (47).

HTST has shown a reduction between 2.4 to more than 4.9-log_10_ for *Staphylococcus aureus* (ATCC 6538) and an inactivation higher than 5.15-log_10_ for *Staphylococcus aureus* (ATCC 25923) at the conditions shown in Table 3. For *Enterococcus faecalis* (ATCC 29212), HTST can achieve an inactivation between 1.1 and higher than 5.1-log_10_, depending on the conditions described in Table 3. After HTST, *Escherichia coli* (ATCC 25922) and *Escherichia coli* (CIVO.B.0505) decreased by 6.07 and 32-log_10_, respectively. HTST can also inactivate CMV and reduce it by more than 2-log_10_ at conditions specified in Table 3 (50, 55–57).

It can be concluded that Holder Pasteurization and HTST (48, 50, 53, 55, 57) were able to achieve the previously mentioned required conditions for microbiological safety in HMB for *Enterococcus faecalis* (ATCC 29212), *Enterococcus* spp., *Escherichia coli* (ATCC 25922), *Staphylococcus aureus* (ATCC 25923), *Staphylococcus aureus* (ATCC 6538), and Cytomegalovirus.

### Effect of thermal processes on breast milk functional components

As shown in Table 4, HoP could only retain between 10% and 20% of lactoferrin’s content, offering high retention (40%–60%) in some cases. On the other hand, lysozyme concentration and activity showed retentions of between 35% and 85%, while Giribaldi et al. achieved complete retention (56). Other authors (Table 4) have reported a 66% mean retention of IgA content, and a similar retention, 61%, of IgG content. Crude protein and carbohydrate content were not affected after this treatment, while only 0%–1.5% of BSSL activity was retained after the HoP application (48, 49, 56, 58–63, 65–69).

Similarly, HTST could retain 32% of lactoferrin’s content, as shown in Table 4. Lysozyme concentration and activity were retained by 72% and 97%, respectively, while IgA and IgG content reported a mean of 84% and 70% of retention, respectively. The study did not report the crude protein and carbohydrate content. Like the results observed in HoP, almost none of the BSSL activity was retained, which confirms the thermosensitivity of this molecule (48, 56, 57). In contrast, another study indicated that gangliosides remain unaffected after HTST treatment. This suggests that gangliosides do not exhibit thermal lability (32).

In general, thermal technologies that use heat flow to increase temperature as a mechanism for microorganism inactivation show a very low ability to retain bioactive components, specifically proteins, such as lactoferrin, lysozyme, and BSSL.

Even though the data show that some improvements in methodology can increase the retention of relevant compounds in HBM, the conclusion is solid: the higher the temperature associated with more extensive time lapses, the lower the retention of bioactive components in HBM.

## Non-thermal processes for breast milk treatment

### High-Pressure Processing (HPP)

High-Pressure Processing (also known as ultrahigh-pressure processing and high hydrostatic pressure processing) is a novel technology that assures food safety and retains quality. As an alternative to traditional thermal food preservation methods, HPP can produce pasteurized products without the extensive loss of quality often associated with heating. The process uses high hydrostatic pressures, typically 100 to 1,000 MPa, and can be combined with heat for brief durations (80, 81). HPP can extend a product’s shelf life and guarantee its safety upon consumption by inactivating enzymes, pathogens, and spoilage organisms at room temperature (80, 81).

Unlike thermal methods, foods treated with HPP often have superior acceptance rates and enhanced sensory quality. This is attributed mainly to HPP’s gentle nature (it primarily affects only weak chemical bonds, thereby preserving vital components related to color, flavor, and nutrition). Additionally, many proteins, vitamins, and bioactive compounds crucial for infants (providing vital properties, such as protection against pathogens, strengthening the microbiota, and fostering proper intestinal development) are barostable, thus unaffected by this process (63, 64, 80, 82).

This method consists, in general, of three steps: (1) the time required to reach the desired pressure (come-up time -CUT-), (2) the period while desired pressure is maintained, called holding time, and (3) the stage when pressure is withdrawn, called depressurization. This technique operates under the following guiding physical principles: (1) Le Chatelier’s principle, which states that if pressure undergoes any change, the process will turn its direction to minimize the effects in the equilibrium state, affecting volume; in practical terms, increasing pressure intensifies any processes associated with volume reduction, (2) the isostatic principle that postulates that pressure acts equally in all directions when it is uniformly applied, meaning that pressure and its effect is immediately and homogenously distributed independently of the size and shape of the sample, (3) the principle of microscopic ordering, which says that the degree of molecular organization of a given substance rises with increasing pressure at constant temperature; as a result, pressure and temperature could have antagonistic effects in food treatment (83), and (4) the Arrhenius relationship, which, similar to thermal processing, states that some reaction speeds during HPP are also influenced by thermal effects. The overall pressure-thermal effects may be additive, antagonistic, or synergistic. It becomes relevant to recognize that hydrostatic pressure modifies interatomic distances, impacting interactions contingent on bond lengths (80, 81).

### UV radiation

UV radiation offers a non-thermal alternative for pasteurization, particularly suitable for HBM. This method uses wavelengths between 100 and 400 nm, divided into three categories based on their physicochemical attributes and biological impacts: UV-A (315 nm to 400 nm), UV-B (280 nm to 315 nm), and UV-C (200 nm to 280 nm) (84). The principle behind this cost-effective process is the germicidal effectiveness of ultraviolet light, which when applied appropriately can penetrate the fluid. The key factors influencing its efficiency are power, wavelength and treatment duration. Although UV-C has limited ability to penetrate dense and opaque liquids, adjusting the flow rate can enhance the process in terms of deactivating microorganisms. Furthermore, creating turbulent flow can result in reduced microbial load (85). Its application aids in extending the shelf life of perishable foods, with UV-C predominantly utilized in food and medical industries due to its pronounced disinfecting properties (72).

Thus, while UV-C radiation has established its relevance in food and medicine, its application to HBM necessitates a thorough evaluation. Striking the right balance between disinfection and nutrient preservation is pivotal for optimizing both the safety and nutritional value intended for infant nutrition (86).

### Pulsed Electric Fields (PEF)

PEF technology presents a novel non-thermal technique for pasteurization with specific implications for HBM. By applying short, high-intensity electric pulses to the targeted sample, PEF alters the permeability of microorganisms’ cell membranes, effectively leading to their inactivation (87).

One of its notable strengths is in preserving the milk’s sensory properties of color and flavor, as well as its nutritional composition (88), which is attributed mainly to its reduced reliance on heat compared to traditional pasteurization techniques.

However, a pertinent consideration with PEF is the potential and inadvertent temperature increases within the milk due to potential ohmic heating effects. While PEF is fundamentally a non-thermal process, incidental ohmic heating effects could trigger lipid oxidation, undermining milk quality and nutritional value (87). Furthermore, PEF has been observed to affect the structure of proteins and amino acids, particularly those linked by weaker bonds, such as disulfide bonds, hydrogen bonds, and hydrophobic interactions (89). More specifically, PEF tends to promote the formation of disulfide bonds, the primary covalent linkages appearing in protein aggregates as an outcome of this treatment. PEF can influence protein structures through hydrophobic interactions and thiol or disulfide reactions, altering protein thermal stability and susceptibility to enzymatic degradation (90).

### Effect of non-thermal processes on breast milk microbiological load

HPP has shown the ability to decrease by 5-log to 8.1-log the species of *Staphylococcus aureus* (ATCC 25923), *Staphylococcus aureus* (ATCC 6538), and *Staphylococcus aureus* sub. *aureus*. *Escherichia coli* (ATCC 25922) showed reductions by 6-log according to data reported in Table 5 (47, 53, 70, 71). HPP can also decrease virus amounts, with Cytomegalovirus and Hepatitis A dropping by 0.9-log and 4-log, respectively (49).

The effect of UV-radiation has been studied on bacteria like *Staphylococcus* (in the forms of *Staphylococcus aureus* (138-CPS and 146-CPS), *Staphylococcus aureus* (ATCC 6538), *Staphylococcus aureus* (PCM 2054), *Staphylococcus epidermis* (ATCC 12228)), achieving reductions between 5-log to complete inactivation under the conditions shown on Table 5 (52, 60, 68, 72). *Enterococcus faecium* (ATCC 6057) has been studied for this technology, showing a decrease of 3.95-log. Also, UV radiation has shown the ability to decrease levels of *Escherichia coli* (K-12) to below-detection. No data about virus inactivation was found for UV-radiation technology (52, 72).

Even though research regarding PEF as a treatment for microorganism inactivation in HBM is scarce, according to data reported in Table 5 (65), this technology can reduce *endogenous bacteria* in HBM from 0.42-log to 4.67-log at the specified conditions in the previously mentioned table.

It can be concluded that HPP (49, 53, 70, 71) was able to achieve the previously mentioned required conditions for microbiological safety of HBM for Hepatitis A virus, *Listeria monocytogenes* (ATCC 19115), *Staphylococcus aureus* (ATCC 25923), and *Staphylococcus aureus* (ATCC 6538). Similarly, UV radiation (52, 60, 72), according to the reviewed data, could achieve these conditions for *Escherichia coli* K 12 (ATCC1498), *Listeria monocytogenes* (ScottA, OSY-428, Ohio, California, ATCC 19115), *Staphylococcus aureus* (138-CPS and 146-CPS), *Staphylococcus aureus* (ATCC 6538), *Staphylococcus aureus* (PCM 2054), *Staphylococcus epidermis* (ATCC 12228).

Even though PEF showed the potential to reduce endogenous bacterial load on HBM, not enough reported data were found to conclude definitively about the effect of this technology on microorganism inactivation.

Regarding the effect of non-thermal treatments on spore-forming bacteria, there are several works included in Table 5, mainly evaluating the effect of HPP, and UV radiation on *B. cereus*, *Paenibacillus macerans* and *Panibacillus polymixa* spores. In (47) and (70), authors describe HPP as more effective than HoP on *B. cereus*. Particularly (47) reported 593.96 MPa for 233 s as the best process conditions. The effect of UV-C treatment is described in (60), achieving a 5-log_10_ reduction in *B. subtilis*, *Paenibacillus macerans*, and *Paenibacillus polymyxa* spores, preserving BSSL activity (Table 6). UV-C’s effectiveness against *B. cereus* spores, maintaining BSSL and fatty acid profiles is described by Christen et al. (68).

### Effect of non-thermal processes on breast milk functional components

Regarding non-thermal technologies, HPP has shown an average of 98% retention of lysozyme activity, achieving complete retention (100%) at 400 MPa, 5 min, 25°C, and 400 MPa, 30 min. The lactoferrin retention varied from 55% to 87%, achieving higher retention at 400 MPa, 5 min, 25°C. IgA content was retained between 70% and 98%, showing a higher retention at 400 MPa, 5 min, 25°C. On the other hand, an average pressure of 600 MPa showed a decrease of IgG by 70%, but preserving ~80% of IgG content at 200 MPa, 10 min; interval 10 min; 400 MPa, 10 min, 19–21°C. The carbohydrate and crude protein did not change after any reported treatment condition. Bile Salt Stimulated Lipase (BSSL) activity showed a 100% retention at 500 MPa, 8 min, 4°C and 400 MPa, 5 min, 25°C conditions, but showing a reduction of almost 40% at 550 MPa, 5 min, according to data shown in Table 6 (49, 58–63, 67).

On the other hand, applying UV radiation to HBM requires careful consideration due to its potential impact on the nutritional composition of the milk. Prolonged UV exposure has diminished vital components like vitamin C, lysozyme, and other essential bioactive compounds and nutrients essential for infant well-being (52). For example, Table 6 shows that BSSL activity had retention levels from 20% to almost 100%, with higher conservation levels at lower radiation doses (253.7 nm, 1.1 W, 4863 J/L, for example) (60, 72).

For PEF, the highest retention levels of IgA and lactoferrin content reported were achieved at 15 kV, 6,000 pulses, and 20 Hz. It is relevant to mention that an area of opportunity to investigate this technology applied to the treatment of HBM was detected (65).

To summarize, the most studied non-thermal technology for Breast Milk treatment, according to reviewed data, is HPP, which has shown promising conservation levels (reaching even 100%) of bioactive and nutritional components in HBM. On the other hand, an opportunity area for research was detected based on the need for more data regarding UV radiation and PEF technologies for HBM treatment.

## Conclusion

Human Breast Milk (HBM) is universally acknowledged as the optimal source of nutrition for neonates due to its rich nutritional content and potential therapeutic properties. However, despite universal endorsement, breastfeeding rates in the US show a decline during an infant’s initial 6 months. This trend highlights the urgent need for reliable methods to provide HBM to infants consistently. Emerging research focuses on preserving techniques that maintain the nutritional richness of HBM without compromising its bioactive components. We described in detail the cutting-edge applications of thermal and non-thermal processes for HBM preservation, emphasizing their impact on reducing pathogenic microorganisms while retaining essential nutrients, enzymes, and immunity properties. Data pointed out that, as expected, thermal technologies such as HoP and HTST can efficiently inactivate common bacteria and microorganisms in HBM but compromise the content and activity of bioactive and nutritional compounds, leading to an opportunity for non-thermal technologies, such as HPP and UV radiation which, according to analyzed data, showed the capacity to achieve conditions for microbiological safety established for HMB while preserving relevant components for newborns. Still, certain areas need further research. The effects of preservation treatments on the carbohydrate composition, especially HMOs and lipids, a vital newborn energy source, are still underexplored. Additionally, the combined use of non-thermal and thermal preservation techniques presents a promising area of study, particularly in terms of their effects on the nutritional and bioactive components of HBM. Understanding how these combined methods can optimize the preservation of HBM while maintaining its essential components is vital, Furthermore, there are significant challenges in determining the post-preservation shelf life of HBM and the effects of preservation on protein denaturation and digestibility. These aspects are critical for newborn gut development and overall health. Ultimately, advancements in HBM preservation serve a twofold purpose: they prioritize infant health and well-being while empowering mothers throughout their lactation journey, regardless of external challenges, for a healthier society where every newborn has access to proper nutrition.

## Author contributions

AN-D: Data curation, Formal analysis, Investigation, Visualization, Writing – original draft. VM-C: Data curation, Investigation, Visualization, Writing – original draft. JW-C: Supervision, Writing – review & editing. SM-Q: Data curation, Investigation, Visualization. CC-H: Conceptualization, Supervision, Writing – review & editing.

## References

[1] Zhang S Li T Xie J Zhang D Pi C Zhou L. Gold standard for nutrition: a review of human milk oligosaccharide and its effects on infant gut microbiota. Microb Cell Factories. (2021) 20:108. doi: 10.1186/s12934-021-01599-y, PMID: 34049536 PMC8162007

[2] Adamkin DH. Use of human milk and fortification in the NICU. J Perinatol. (2023) 43:551–9. doi: 10.1038/s41372-022-01532-0, PMID: 36257977

[3] Nuzzi G Di Cicco ME Peroni DG. Breastfeeding and allergic diseases: what’s new? Children. (2021) 8:330. doi: 10.3390/children8050330, PMID: 33923294 PMC8145659

[4] Selma-Royo M Calvo Lerma J Cortés-Macías E Collado MC. Human milk microbiome: from actual knowledge to future perspective. Semin Perinatol. (2021) 45:151450. doi: 10.1016/j.semperi.2021.151450, PMID: 34274151

[5] CDC. Breastfeeding report card | breastfeeding | CDC. (2023). Available at: https://www.cdc.gov/breastfeeding/data/reportcard.htm

[6] United Nations Children’s Fund (UNICEF). Breastfeeding, a mother's gift, for every child. New York City: UNICEF (2018) Available at: https://www.unicef.org/media/48046/file/UNICEF_Breastfeeding_A_Mothers_Gift_for_Every_Child.pdf.

[7] Yan J Liu L Zhu Y Huang G Wang PP. The association between breastfeeding and childhood obesity: a meta-analysis. BMC Public Health. (2014) 14:1267. doi: 10.1186/1471-2458-14-1267 25495402 PMC4301835

[8] Horta BL Loret De Mola C Victora CG. Long-term consequences of breastfeeding on cholesterol, obesity, systolic blood pressure and type 2 diabetes: a systematic review and meta-analysis. Acta Paediatr. (2015) 104:30–7. doi: 10.1111/apa.13133, PMID: 26192560

[9] Bai Y Wunderlich SM. Lactation accommodation in the workplace and duration of exclusive breastfeeding. J Midwifery Womens Health. (2013) 58:690–6. doi: 10.1111/jmwh.12072, PMID: 24325729

[10] World Health Organization. Preterm birth. (2023). Available at: https://www.who.int/news-room/fact-sheets/detail/preterm-birth

[11] Stuebe AM Schwarz EB Grewen K Rich-Edwards JW Michels KB Foster EM. Duration of lactation and incidence of maternal hypertension: a longitudinal cohort study. Am J Epidemiol. (2011) 174:1147–58. doi: 10.1093/aje/kwr227 21997568 PMC3246687

[12] Pacza T Martins ML Rockaya M Müller K Chatterjee A Barabási AL. MilkyBase, a database of human milk composition as a function of maternal-, infant- and measurement conditions. Sci Data. (2022) 9:557. doi: 10.1038/s41597-022-01663-1, PMID: 36085296 PMC9463137

[13] Witkowska-Zimny M Kaminska-El-Hassan E. Cells of human breast milk. Cell Mol Biol Lett. (2017) 22:1–11. doi: 10.1186/s11658-017-0042-4 28717367 PMC5508878

[14] Kaingade P Somasundaram I Nikam A Behera P Kulkarni S Patel J. Breast milk cell components and its beneficial effects on neonates: need for breast milk cell banking. J Pediatr Neonat Individ Med. (2017) 6:e060115. doi: 10.7363/060115

[15] Boquien CY. Human milk: an ideal food for nutrition of preterm newborn. Front Pediatr. (2018) 6:295. doi: 10.3389/fped.2018.00295, PMID: 30386758 PMC6198081

[16] NeoLacta Lifesciences. Different stages of breastmilk composition. (2023). Available at: https://neolacta.com/blogs/different-stages-of-breastmilk-composition-updated/

[17] American Pregnancy Association. Breastfeeding: overview. (2023). Available at: https://americanpregnancy.org/healthy-pregnancy/breastfeeding/breastfeeding-overview/

[18] Andreas NJ Kampmann B Mehring Le-Doare K. Human breast milk: a review on its composition and bioactivity. Early Hum Dev. (2015) 91:629–35. doi: 10.1016/j.earlhumdev.2015.08.013 26375355

[19] Brahm P Valdés V. The benefits of breastfeeding and associated risks of replacement with baby formulas. Rev Chil Pediatr. (2017) 88:07–14. doi: 10.4067/S0370-41062017000100001 28288222

[20] Mosca F Giannì ML. Human milk: composition and health benefits. Pediatr Med Chir. (2017) 39:155. doi: 10.4081/pmc.2017.155, PMID: 28673076

[21] Peila C Moro GE Bertino E Cavallarin L Giribaldi M Giuliani F. The effect of holder pasteurization on nutrients and biologically-active components in donor human milk: a review. Nutrients. (2016) 8:477. doi: 10.3390/nu8080477, PMID: 27490567 PMC4997390

[22] Czosnykowska-Łukacka M Orczyk-Pawiłowicz M Broers B Królak-Olejnik B. Lactoferrin in human milk of prolonged lactation. Nutrients. (2019) 11:2350. doi: 10.3390/nu11102350, PMID: 31581741 PMC6835443

[23] Kaplan M Sahutoglu AS Sarıtas S Duman H Arslan A Pekdemir B. Role of milk glycome in prevention, treatment, and recovery of COVID-19. Front Nutr. (2022) 9:1033779. doi: 10.3389/fnut.2022.1033779, PMID: 36424926 PMC9680090

[24] Bolat E Eker F Kaplan M Duman H Arslan A Saritas S. Lactoferrin for COVID-19 prevention, treatment, and recovery. Front Nutr. (2022) 9:992733. doi: 10.3389/fnut.2022.992733, PMID: 36419551 PMC9676636

[25] Karav S. Selective deglycosylation of lactoferrin to understand glycans’ contribution to antimicrobial activity of lactoferrin. Cell Mol Biol. (2018) 64:52–7. doi: 10.14715/cmb/2018.64.9.8, PMID: 30030954

[26] Minami J Odamaki T Hashikura N Abe F Xiao JZ. Lysozyme in breast milk is a selection factor for bifidobacterial colonisation in the infant intestine. Benef Microbes. (2016) 7:53–60. doi: 10.3920/BM2015.0041, PMID: 26503736

[27] Zivkovic AM German JB Lebrilla CB Mills DA. Human milk glycobiome and its impact on the infant gastrointestinal microbiota. Proc Natl Acad Sci U S A. (2011) 108:4653–8. doi: 10.1073/pnas.1000083107, PMID: 20679197 PMC3063602

[28] Karav S Le Parc A Nobrega L de Moura Bell JM Frese SA Kirmiz N. Oligosaccharides released from milk glycoproteins are selective growth substrates for infant-associated bifidobacteria. Appl Environ Microbiol. (2016) 82:3622–30. doi: 10.1128/AEM.00547-16, PMID: 27084007 PMC4959171

[29] Enam F Mansell TJ. Linkage-specific detection and metabolism of human milk oligosaccharides in *Escherichia coli*. Cell Chem Biol. (2018) 25:1292–1303.e4. doi: 10.1016/j.chembiol.2018.06.002, PMID: 30017916

[30] Brink LR Lönnerdal B. Milk fat globule membrane: the role of its various components in infant health and development. J Nutr Biochem. (2020) 85:108465. doi: 10.1016/j.jnutbio.2020.108465 32758540

[31] Koletzko B. Human milk lipids. Ann Nutr Metab. (2017) 69:27–40. doi: 10.1159/000452819 28103608

[32] Salcedo J Karav S Le Parc A Cohen JL de Moura Bell JMLN Sun A. Application of industrial treatments to donor human milk: influence of pasteurization treatments, storage temperature, and time on human milk gangliosides. NPJ Sci Food. (2018) 2:5. doi: 10.1038/s41538-018-0013-9 31304255 PMC6550147

[33] Walker A. Breast milk as the gold standard for protective nutrients. J Pediatr. (2010) 156:S3–7. doi: 10.1016/j.jpeds.2009.11.021, PMID: 20105662

[34] Binns C Lee M Low WY. The long-term public health benefits of breastfeeding. Asia Pac J Public Health. (2016) 28:7–14. doi: 10.1177/1010539515624964 26792873

[35] Prentice AM. Breastfeeding in the modern world. Ann Nutr Metab. (2022) 78:29–38. doi: 10.1159/000524354 35679837

[36] Su D Pasalich M Lee AH Binns CW. Ovarian cancer risk is reduced by prolonged lactation: a case-control study in southern China. Am J Clin Nutr. (2013) 97:354–9. doi: 10.3945/ajcn.112.044719 23283498

[37] del Ciampo LA del Ciampo IRL. Breastfeeding and the benefits of lactation for women’s health. Rev Bras Ginecol Obstet. (2018) 40:354–9. doi: 10.1055/s-0038-1657766 29980160 PMC10798271

[38] Hajat C Stein E. The global burden of multiple chronic conditions: a narrative review. Prev Med Rep. (2018) 12:284–93. doi: 10.1016/j.pmedr.2018.10.008, PMID: 30406006 PMC6214883

[39] CDC. The surgeon general’s call to action to support breastfeeding | breastfeeding | CDC. (2023). Available at: https://www.cdc.gov/breastfeeding/resources/calltoaction.htm

[40] Escuder-Vieco D Espinosa-Martos I Rodríguez JM Corzo N Montilla A Siegfried P. High-temperature short-time pasteurization system for donor milk in a human milk bank setting. Front Microbiol. (2018) 9:926. doi: 10.3389/fmicb.2018.00926, PMID: 29867837 PMC5958646

[41] Collins A Weitkamp JH Wynn JL. Why are preterm newborns at increased risk of infection? Arch Dis Child Fetal Neonatal Ed. (2018) 103:F391–4. doi: 10.1136/archdischild-2017-313595, PMID: 29382648 PMC6013388

[42] Human Milk Banking Association of North America. HMBANA standards for donor human milk banking: an overview HMBANA guidelines committee HMBANA standards for donor human milk banking: an overview. (2023). Available at: www.hmbana.orginfo@hmbana.org

[43] Thomas CL Murphy LD Mills MJ Zhang J Fisher GG Clancy RL. Employee lactation: a review and recommendations for research, practice, and policy. Hum Resour Manag Rev. (2022) 32:100848. doi: 10.1016/j.hrmr.2021.100848

[44] Tsai SY. Impact of a breastfeeding-friendly workplace on an employed mother’s intention to continue breastfeeding after returning to work. Breastfeed Med. (2013) 8:210–6. doi: 10.1089/bfm.2012.0119, PMID: 23390987 PMC3616406

[45] Manzardo OA Toll LJ Müller K Nickel E Jonas D Baumgartner J. A novel heat treatment protocol for human milk. Front Pediatr. (2022) 10:990871. doi: 10.3389/fped.2022.990871, PMID: 36330365 PMC9623327

[46] Calvo J García Lara NR Gormaz M Peña M Martínez Lorenzo MJ Ortiz Murillo P. Recommendations for the creation and operation of maternal milk banks in Spain. An Pediatr. (2018) 89:65.e1–6. doi: 10.1016/j.anpede.2018.01.007, PMID: 29496426

[47] Rocha-Pimienta J Martillanes S Ramírez R Garcia-Parra J Delgado-Adamez J. *Bacillus cereus* spores and *Staphylococcus aureus* sub. Aureus vegetative cells inactivation in human milk by high-pressure processing. Food Control. (2020) 113:107212. doi: 10.1016/j.foodcont.2020.107212

[48] Klotz D Joellenbeck M Winkler K Kunze M Huzly D Hentschel R. High-temperature short-time pasteurisation of human breastmilk is efficient in retaining protein and reducing the bacterial count. Acta Paediatr Int J Paediatr. (2017) 106:763–7. doi: 10.1111/apa.13768, PMID: 28135766

[49] Pitino MA Unger S Gill A McGeer AJ Doyen A Pouliot Y. High pressure processing inactivates human cytomegalovirus and hepatitis a virus while preserving macronutrients and native lactoferrin in human milk. IFSET. (2022) 75:102891. doi: 10.1016/j.ifset.2021.102891

[50] Klotz D Schreiner M Falcone V Jonas D Kunze M Weber A. High-temperature short-time treatment of human milk for bacterial count reduction. Front Pediatr. (2018) 6:359. doi: 10.3389/fped.2018.00359 30538974 PMC6277678

[51] Bouquet P Alexandre V De Lamballerie M Ley D Lesage J Goffard A. Effect of high hydrostatic pressure processing and holder pasteurization of human milk on inactivation of human coronavirus 229e and hepatitis e virus. Viruses. (2023) 15:1571. doi: 10.3390/v15071571, PMID: 37515257 PMC10384040

[52] Martysiak-Żurowska D Puta M Kotarska J Cybula K Malinowska-Pańczyk E Kołodziejska I. The effect of UV-C irradiation on lipids and selected biologically active compounds in human milk. Int Dairy J. (2017) 66:42–8. doi: 10.1016/j.idairyj.2016.10.009

[53] Viazis S Farkas BE Jaykus LA. Inactivation of bacterial pathogens in human milk by high-pressure processing. J Food Prot. (2008) 71:109–18. doi: 10.4315/0362-028X-71.1.109, PMID: 18236670

[54] Lackey KA Pace RM Williams JE Bode L Donovan SM Järvinen KM. SARS-CoV-2 and human milk: what is the evidence? Matern Child Nutr. (2020) 16:e13032. doi: 10.1111/mcn.13032 32472745 PMC7300480

[55] Terpstra FG Rechtman DJ Lee ML Van Hoeij K Berg H Van Engelenberg FAC. Antimicrobial and antiviral effect of high-temperature short-time (HTST) pasteurization applied to human milk. Breastfeed Med. (2007) 2:27–33. doi: 10.1089/bfm.2006.0015, PMID: 17661617

[56] Giribaldi M Coscia A Peila C Antoniazzi S Lamberti C Ortoffi M. Pasteurization of human milk by a benchtop high-temperature short-time device. IFSET. (2016) 36:228–33. doi: 10.1016/j.ifset.2016.07.004

[57] Dhar J Fichtali J Skura BJ Nakai S Davidson AGF. Pasteurization efficiency of a HTST system for human milk. J Food Sci. (1996) 61:569–73. doi: 10.1111/j.1365-2621.1996.tb13160.x

[58] Wesolowska A Sinkiewicz-Darol E Barbarska O Strom K Rutkowska M Karzel K. New achievements in high-pressure processing to preserve human milk bioactivity. Front Pediatr. (2018) 6:323. doi: 10.3389/fped.2018.00323, PMID: 30519550 PMC6250976

[59] Pitino MA Unger S Doyen A Pouliot Y Aufreiter S Stone D. High hydrostatic pressure processing better preserves the nutrient and bioactive compound composition of human donor milk. J Nutr. (2019) 149:497–504. doi: 10.1093/jn/nxy302, PMID: 30770541 PMC6398389

[60] Koh J Victor AF Howell ML Yeo JG Qu Y Selover B. Bile salt-stimulated lipase activity in donor breast milk influenced by pasteurization techniques. Front Nutr. (2020) 7:552362. doi: 10.3389/fnut.2020.552362, PMID: 33282897 PMC7689290

[61] Zhang J Lee NA Duley JA Cowley DM Shaw PN Bansal N. Comparing the effects of hydrostatic high-pressure processing vs holder pasteurisation on the microbial, biochemical and digestion properties of donor human milk. Food Chem. (2022) 373:131545. doi: 10.1016/j.foodchem.2021.131545, PMID: 34839967

[62] Dussault N Cayer MP Landry P De Grandmont MJ Cloutier M Thibault L. Comparison of the effect of holder pasteurization and high-pressure processing on human milk bacterial load and bioactive factors preservation. J Pediatr Gastroenterol Nutr. (2021) 72:756–62. doi: 10.1097/MPG.0000000000003065, PMID: 33847290 PMC8549451

[63] Viazis S Farkas BE Allen JC. Effects of high-pressure processing on immunoglobulin a and lysozyme activity in human milk. J Hum Lact. (2007) 23:253–61. doi: 10.1177/0890334407303945

[64] Rogier EW Frantz AL Bruno MEC Wedlund L Cohen DA Stromberg AJ. Secretory antibodies in breast milk promote long-term intestinal homeostasis by regulating the gut microbiota and host gene expression. Proc Natl Acad Sci U S A. (2014) 111:3074–9. doi: 10.1073/pnas.1315792111, PMID: 24569806 PMC3939878

[65] Zhang J Ghasemi N Zare F Duley JA Cowley DM Shaw PN. Nanosecond pulsed electric field treatment of human milk: effects on microbiological inactivation, whey proteome and bioactive protein. Food Chem. (2023) 406:135073. doi: 10.1016/j.foodchem.2022.135073, PMID: 36455315

[66] Buffin R Hays S Drai J Sarda MN Picaud JC. Better control of holder pasteurization results in higher retention of human milk lactoferrin, IgA, and lysozyme. Front Pediatr. (2018) 6:381. doi: 10.3389/fped.2018.00381 30560111 PMC6287107

[67] Permanyer M Castellote C Ramírez-Santana C Audí C Pérez-Cano FJ Castell M. Maintenance of breast milk immunoglobulin a after high-pressure processing. J Dairy Sci. (2010) 93:877–83. doi: 10.3168/jds.2009-2643, PMID: 20172207

[68] Christen L Lai CT Hartmann B Hartmann PE Geddes DT. Ultraviolet-C irradiation: a novel pasteurization method for donor human milk. PLoS One. (2013) 8:e68120. doi: 10.1371/journal.pone.0068120, PMID: 23840820 PMC3694044

[69] Lima HK Wagner-Gillespie M Perrin MT Fogleman AD. Bacteria and bioactivity in holder pasteurized and shelf-stable human milk products. Curr Dev Nutr. (2017) 1:e001438. doi: 10.3945/cdn.117.001438, PMID: 29955718 PMC5998364

[70] Demazeau G Plumecocq A Lehours P Martin P Couëdelo L Billeaud C. A new high hydrostatic pressure process to assure the microbial safety of human milk while preserving the biological activity of its main components. Front Public Health. (2018) 6:306. doi: 10.3389/fpubh.2018.00306, PMID: 30460221 PMC6232532

[71] Windyga B Rutkowska M Sokołowska B Skąpska S Wesołowska A Wilińska M. Inactivation of Staphylococcus aureus and native microflora in human milk by high pressure processing. High Press Res. (2015) 35:181–8. doi: 10.1080/08957959.2015.1007972

[72] Christen L Lai CT Hartmann B Hartmann PE Geddes DT. The effect of UV-C pasteurization on bacteriostatic properties and immunological proteins of donor human milk. PLoS One. (2013) 8:e85867. doi: 10.1371/journal.pone.0085867 24376898 PMC3871660

[73] Capriati T Goffredo BM Argentieri M De Vivo L Bernaschi P Cairoli S. A modified holder pasteurization method for donor human milk: preliminary data. Nutrients. (2019) 11:1139. doi: 10.3390/nu11051139, PMID: 31121859 PMC6566761

[74] Caballero Martín S Sánchez Gomez de Orgaz MC Sánchez Luna M. Estudio de calidad de la pasteurización Holder de leche materna donada en una unidad de nutrición personalizada neonatal. An Pediatr. (2022) 96:294–9. doi: 10.1016/j.anpedi.2021.01.019

[75] Gómez de Segura A Moles L Montilla A Corzo N Leónides Fernández Rodríguez Juan M. Efecto de la pasteurización Holder sobre parámetros microbiológicos, inmunológicos y bioquímicos en muestras de leche humana donada. Digital CSIC (2010).

[76] Meunier-Goddik L Sandra S. Liquid milk products: pasteurized milk. In: WF John, editor. Encyclopedia of dairy sciences. 2nd edn. Academic Press (2011). 274–80.

[77] Kontopodi E Boeren S Stahl B van Goudoever JB van Elburg RM Hettinga K. High-temperature short-time preserves human milk’s bioactive proteins and their function better than pasteurization techniques with long processing times. Front Pediatr. (2022) 9:9. doi: 10.3389/fped.2021.798609 PMC8811466 35127595

[78] Ih M Mather IH Keenan TW. Milk fat globule membrane In: Encyclopedia of dairy science: Elsevier (2011). 680–90.

[79] Escuder-Vieco D Espinosa-Martos I Rodríguez JM Fernández L Pallás-Alonso CR. Effect of htst and holder pasteurization on the concentration of immunoglobulins, growth factors, and hormones in donor human milk. Front Immunol. (2018) 9:2222. doi: 10.3389/fimmu.2018.02222 30319659 PMC6170621

[80] Serna-Hernandez SO Escobedo-Avellaneda Z García-García R Rostro-Alanis De J Welti-Chanes J. High hydrostatic pressure induced changes in the physicochemical and functional properties of milk and dairy products: a review. Foods. (2021) 10:1867. doi: 10.3390/foods10081867, PMID: 34441644 PMC8391368

[81] Balasubramaniam VMB Martínez-Monteagudo SI Gupta R. Principles and application of high pressure-based technologies in the food industry. Ann Rev Food Sci and Technol. (2015) 6:435–62. doi: 10.1146/annurev-food-022814-015539, PMID: 25747234

[82] Valencia-Flores DC Hernández-Herrero M Guamis B Ferragut V. Comparing the effects of ultra-high-pressure homogenization and conventional thermal treatments on the microbiological, physical, and chemical quality of almond beverages. J Food Sci. (2013) 78:E199–205. doi: 10.1111/1750-3841.12029, PMID: 23363294

[83] Balny C Masson P. Effects of high pressure on proteins. Food Rev Int. (1993) 9:611–28. doi: 10.1080/87559129309540980

[84] Bintsis T Litopoulou-Tzanetaki E Robinson RK. Review existing and potential applications of ultraviolet light in the food industry-a critical review. J Sci Food and Agric. (2000) 80:637–45. doi: 10.1002/(SICI)1097-0010(20000501)80:6<637::AID-JSFA603>3.0.CO;2-1, PMID: 29345786

[85] Morales de la Peña M Welti-Chanes J Martín-Belloso O. Novel technologies to improve food safety and quality. Curr Opin Food Sci. (2018) 30:1–7. doi: 10.1016/j.cofs.2018.10.009

[86] Kontopodi E Stahl B van Goudoever JB Boeren S Timmermans RAH den Besten HMW. Effects of high-pressure processing, UV-C irradiation and thermoultrasonication on donor human milk safety and quality. Front Pediatr. (2022) 10:828448. doi: 10.3389/fped.2022.828448, PMID: 35386262 PMC8979557

[87] Mohamad A Shah NNAK Sulaiman A Mohd Adzahan N Aadil RM. Impact of the pulsed electric field on physicochemical properties, fatty acid profiling, and metal migration of goat milk. J Food Process Preserv. (2020) 44:e14940. doi: 10.1111/jfpp.14940

[88] Ahmad T Butt MZ Aadil RM Inam-ur-Raheem M Abdullah Bekhit AED. Impact of nonthermal processing on different milk enzymes. Int J Dairy Technol. (2019) 72:481–95. doi: 10.1111/1471-0307.12622

[89] Zhao W Yang R. Pulsed electric field induced aggregation of food proteins: ovalbumin and bovine serum albumin. Food Bioprocess Technol. (2012) 5:1706–14. doi: 10.1007/s11947-010-0464-8

[90] Buckow R Chandry PS Ng SY McAuley CM Swanson BG. Opportunities and challenges in pulsed electric field processing of dairy products. Int Dairy J. (2014) 34:199–212. doi: 10.1016/j.idairyj.2013.09.002

